# Advances in CAR-T Cell Genetic Engineering Strategies to Overcome Hurdles in Solid Tumors Treatment

**DOI:** 10.3389/fimmu.2022.830292

**Published:** 2022-02-08

**Authors:** Alain E. Andrea, Andrada Chiron, Sarah Mallah, Stéphanie Bessoles, Guillaume Sarrabayrouse, Salima Hacein-Bey-Abina

**Affiliations:** ^1^ Laboratoire de Biochimie et Thérapies Moléculaires, Faculté de Pharmacie, Université Saint Joseph de Beyrouth, Beirut, Lebanon; ^2^ Université de Paris, CNRS, INSERM, UTCBS, Unité des technologies Chimiques et Biologiques pour la Santé, Paris, France; ^3^ Clinical Immunology Laboratory, Groupe Hospitalier Universitaire Paris-Sud, Hôpital Kremlin-Bicêtre, Assistance Publique-Hôpitaux de Paris, Le-Kremlin-Bicêtre, France; ^4^ Faculty of Arts and Sciences, Lebanese American University, Beirut, Lebanon

**Keywords:** CAR-T cell immunotherapy, Tumor microenvironment, Solid tumor, Tumor Homing, Chemokines, Angiogenesis, Tumor stroma, Immune checkpoints

## Abstract

During this last decade, adoptive transfer of T lymphocytes genetically modified to express chimeric antigen receptors (CARs) emerged as a valuable therapeutic strategy in hematological cancers. However, this immunotherapy has demonstrated limited efficacy in solid tumors. The main obstacle encountered by CAR-T cells in solid malignancies is the immunosuppressive tumor microenvironment (TME). The TME impedes tumor trafficking and penetration of T lymphocytes and installs an immunosuppressive milieu by producing suppressive soluble factors and by overexpressing negative immune checkpoints. In order to overcome these hurdles, new CAR-T cells engineering strategies were designed, to potentiate tumor recognition and infiltration and anti-cancer activity in the hostile TME. In this review, we provide an overview of the major mechanisms used by tumor cells to evade immune defenses and we critically expose the most optimistic engineering strategies to make CAR-T cell therapy a solid option for solid tumors.

## 1 Introduction

Chimeric antigen receptor (CAR)-T cells are genetically engineered T lymphocytes with an extracellular antibody-like domain (consisting of a single chain variable fragment or scFv), a transmembrane domain and an intracellular signaling domain. Four main generations of CAR-T cells have been designed to date. The main driver of genetically engineered enhancements across all these generations is the improvement of the anticancer potential of this innovative immunotherapy. First-generation CAR-T cells are engineered with a single activating intracellular domain, CD3ζ, (known as signal 1), without any additional costimulatory domains. As these CAR-T cells cannot produce enough interleukin (IL)-2 –vital for proliferation and growth- exogenous administration of IL-2 (IL-2 immunotherapy) is necessary to enhance CAR-T cells persistence *in vivo* and, thus, anticancer activity. Second- and third-generation CAR-T cells are genetically engineered with one or more intracellular costimulatory domains (known as signal 2), which increases CAR-T cell efficacy and persistence (1). In the case of fourth-generation CAR-T cells, also known as T cell redirected for antigen-unrestricted cytokine-initiated killing (TRUCKs), an additional cassette coding for a transgenic protein (such as a cytokine) is expressed. This protein is released by the genetically modified lymphocytes and modulates their anti-cancer response (2).

Adoptive transfer of CAR-T cells has shown immense success in treating B cell malignancies. In the contrary, the response rates of CAR-T cell immunotherapy among solid cancer patients are less favorable. Major obstacles in solid tumor immunotherapy with CAR-T cells are, first, difficulties in tumor targeting and second an insufficient trafficking and fitness of genetically modified T cells, especially in the hostile tumor microenvironment (TME). Because of the lack of tumor-specific antigens (TSA) or the heterogeneous expression of tumor associated antigens (TAA) with overlapping expression between healthy tissues and tumor cells, one of the roadblocks to effective CAR-T immunotherapy is specific tumor targeting. Hurdles in solid tumor targeting make it a challenge to develop safe immunotherapies devoid of on-target/off-tumor toxicities. Moreover, TAAs can be lost in case of tumor antigen escape (as the case of proliferating tumor subclones), with CAR-T cell immunotherapy becoming ineffective. Other drawbacks, some inherent to CAR-T cells, are represented by limited tumor trafficking and tumor infiltration, as well as an insufficient expansion and persistence of genetically modified T cells in the homeostatic cytokine-deprived TME. All these challenges have been addressed by various preclinical models recently and efforts to improve engineering are still ongoing. In this review, we expose the major obstacles that CAR-T cells face in solid tumors, especially the decrease of T lymphocytes infiltration to the tumor site, the immunosuppressive milieu and the inhibition of CAR-T cell activity by the negative immune checkpoints, and we propose, by reviewing the literature, an extensive list of solutions to each of the mentioned obstacles.

## 2 Challenges and Engineering Strategies to Overcome CAR-T Cells’ Limitations in Solid Tumors

### 2.1 Enhancing CAR-T Cells Tumor Trafficking and Penetration

Solid tumors are organ-like, disorganized structures composed of proliferating tumor cells surrounded by supporting stromal cells and by nourishing blood vessels of the tumor neovasculature and associated to a cellular immune infiltrate composed of both innate and adaptive immune cells. Tumor growth can be controlled by both the innate and adaptive components of the immune system. Therefore, the infiltrating cell populations in solid tumors are comprised of both innate immune cells: neutrophils, macrophages, dendritic cells (DCs), mast cells, natural killer cells (NK cells), and myeloid derived suppressor cells (MDSCs) and of adaptive immune cells: T and B lymphocytes, and regulatory T cells (Tregs). All these immune cells are associated with non-tumor-stromal cells composing the TME: endothelial cells, fibroblasts, pericytes, and mesenchymal cells. All these cells, as well as their secreted factors and molecules compose the TME, an immunosuppressive, hostile milieu for tumor-infiltrating T cells (TILs) and a physical barrier for T cell migration and tumor infiltration.

Among all aforementioned cells, the key player of the anti-tumor response are TILs, their capacity to infiltrate the tumor bed being related to tumor outgrowth and extension (3–5). It is well acknowledged that TILs are a trademark of ongoing tumor immunosurveillance as they have shown both therapeutic and prognostic significance in animals and in humans. Indeed, higher density of TILs in patients’ TME correlates with improved clinical outcomes (6), whereas fail to respond to immunotherapy is associated with a low post-treatment infiltration of T cells (7).

Therefore, the key for successful therapeutic strategies is the switch from a poorly infiltrated “cold” TME or from an “immune-excluded” TME [i.e limited presence of T cells at the periphery of tumor nests without intra-tumoral infiltration (8)] to a “hot” TME, with a rich, active, immune cell infiltrate in the tumor core, especially including functional TILs (9).

Despite initial expectations in solid tumor treatment with CAR-T cell therapies, one major roadblock in treating solid tumors turned out to be the limited access of cellular therapies to the tumor bed, as T cells must face additional barriers before inducing their antitumor activity (10). Indeed, great response to systemically infused CAR-T cells in hematological cancers is due, at least in part, to the easy access of CAR-T cells to malignant cells residing in hematologic organs readily accessible to the blood flow (bone marrow, lymph nodes, spleen) (10).

#### 2.1.1 T cell Trafficking and Homing to Tumor Sites

T cell trafficking to both lymphoid organs and peripheral tissues is tightly regulated by chemotactic cues and controlled by chemokine/chemokine receptors axis and adhesion molecules interactions. T cell migration from the bloodstream and homing into peripheral tissues is a regulated, three-step process starting with 1) an initial transitory attachment and selectin-mediated rolling on the endothelium, followed by 2) chemokine-receptor mediated activation of integrins and finally by 3) integrin-dependent transmigration and extravasation (11, 12). Homing and retention of naïve T cells to lymph nodes is regulated by the expression of CD62L and of the CC chemokine receptor 7 (CCR7, which binds lymph-nodes chemokines CCL19 and CCL21), accompanied by the activation of LFA-1 (13). After T cell priming by antigen presentation, central memory T cells (TCM) lose the expression of both CCR7 and CD62L to acquire an effector memory (CD45RO+) phenotype (TEM), thereby losing their ability to access lymph nodes through the high endothelial venules (HEV).

**Figure 1:**
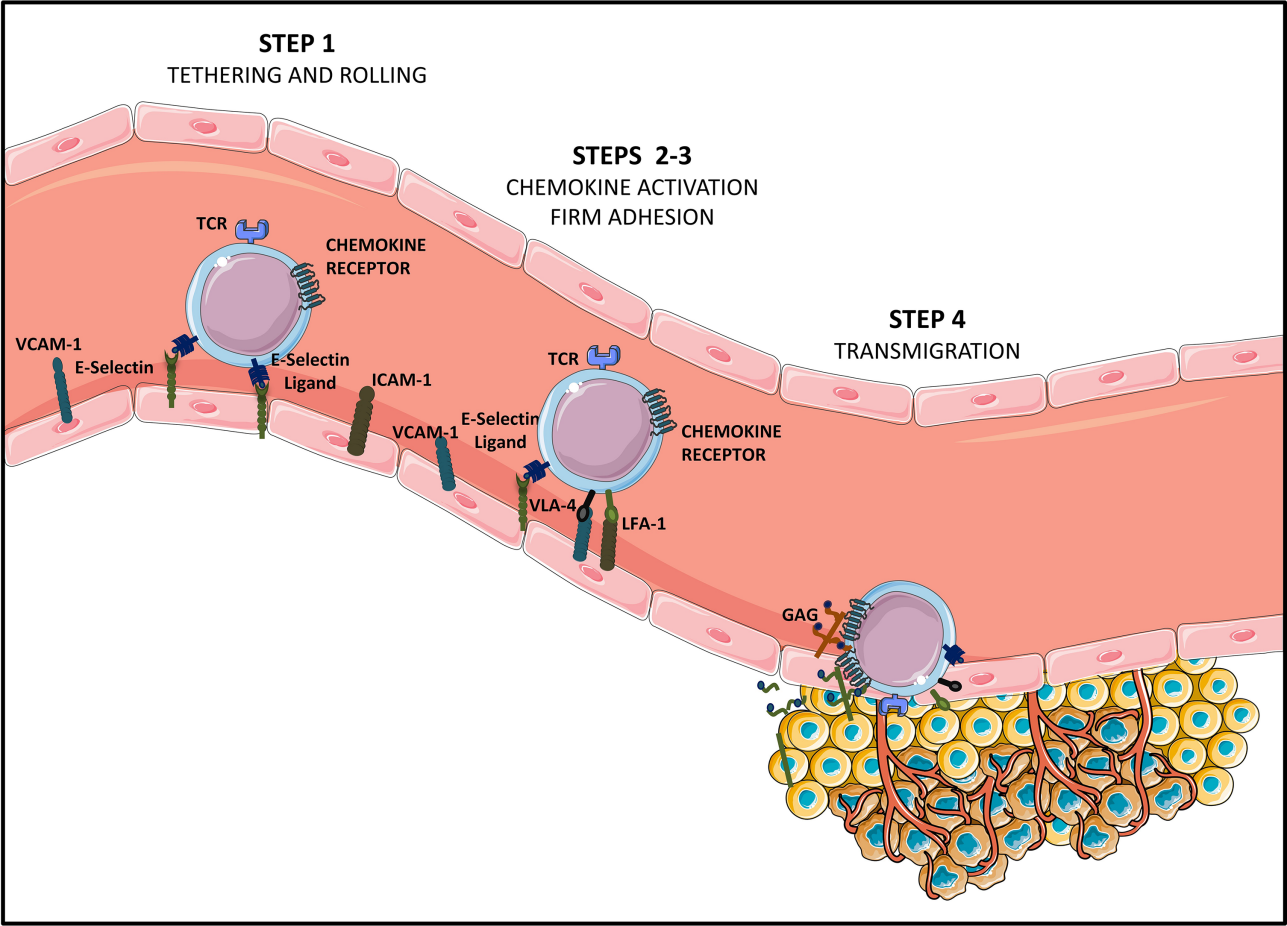
Steps of T cell homing to tumor tissues [Adapted from Sackstein et al. (11)]. Tumor infiltrating CD8+ effector T lymphocytes (Teffs) presenting a specific tumor antigen circulate in the blood stream. They express homing molecules allowing for their oriented migration towards the tumor (like CXCR3 and CCR5-chemokine receptors), as well as ligands allowing binding to endothelial cells (E-selectin ligands and VLA-4 and LFA-1 integrins at suboptimal levels). Circulating Teffs tether and roll on the endothelium (STEP 1) *via* engagement of E-Selectin ligands with endothelial E-Selectin, which slows down Teffs, and allows firm adhesion to the endothelium (STEP 2). In this second step of Teffs entry into tumoral tissues, chemokines produced by cancer cells or by stromal cells from the TME (CXCL9, CXCL10, CCL5…) bind chemokine receptors. This binding of chemokine receptors to their ligands elicits activation of VLA-4 and LFA-1, allowing for VLA-4/VCAM-1 and LFA-1/ICAM-1 firm adhesion (STEP 3). Firmly adherent Teffs undergo transendothelial migration (STEP 4), to infiltrate the TME and establish cell-to-cell contact with tumor cells, *via* TCR-based recognition of cancer antigens presented on HLA molecules.

Therefore, TEMs recirculate in the bloodstream to migrate to peripheral tissues and their migration back to the lymphoid organs is inhibited. Instead, activated T cells gain expression of a cohort of homing molecules that enable them to migrate to diseased/inflamed tissues (14). The T cell effector population presenting with homing capacity to tumor sites expresses homing molecules including ligands for E-selectin (CD62E) and P-selectin (CD62P) expressed on activated endothelial cells as well as chemokine receptors for inflammatory chemokines, such as CXCR3 which binds inflammatory chemokines CXCL9 and CXCL10 (14) and CCR5 which binds respectively to CCL3/CCL3L1/CCL4/CCL5/CCL8/CCL11/CCL13/CCL16 ligands produced by tumor tissues (15, 16). Moreover, the activation of chemokine receptors enables adhesion to the endothelium by inducing the expression of two integrins: β2-integrin leukocyte function-associated antigen-1 (LFA-1), and very late antigen-4 (VLA-4, also known as α4β1), which bind respectively to ICAM-1 and VCAM-1 receptors expressed on the endothelium (17). Upon activation, integrins express binding sites that interact with cell adhesion molecules on the blood vessel walls, leading to T cell firm adhesion and transmigration into the tumor site (18) (**Figure 1**).

Peripheral tissues are the homing site for specialized memory T cell subsets identified and characterized extensively in the context of infectious diseases, called tissue resident memory T cells or TRM, and whose presence in solid cancer is associated with better outcomes. TRMs are localized in non-lymphoid peripheral tissues, do not recirculate and have a unique surface phenotype characterized by the lack of expression of receptors/transcription factors enabling egress from the tissues and lymph node homing (CCR7, CD62L, S1pr1 and Klf2). TRMs express the activation marker CD69, and the integrins CD103 (αEβ7) and CD49a (α1β1), which bind to E-cadherin and type IV collagen on epithelial and endothelial cells, respectively. They also upregulate the LFA1 integrin (αL(CD11a) β2), which binds to the ICAM-1 adhesion molecule on endothelial cells (19). Moreover, CD8+ TILs with a TRM phenotype expressing the adenosine producing ectonucleotidase CD39 and the CD103 integrin are a unique, specific tumor-reactive population found exclusively in the TME, both in primary and metastatic tumors, and whose frequencies are associated with overall survival (OS) in some cancer patients (20). Furthermore, it has recently been shown that a high density of CD8+CD103+CD49a+CD69+ TRM TILs correlates with an improved response to anti-programmed death 1 (PD-1) immune checkpoint blockade (19). Immune checkpoint inhibitors (i.e. ICI) represent a new Nobel-Prize worth immunotherapy with immense success in some incurable cancers, which target s inhibitory costimulatory molecules on the surface of T cells (like PD-1 or CTLA-4).

Barriers limiting access of CAR-T cells to the tumor bed are both physical (represented by surrounding blood vessels and the tumor stroma) and functional (represented by immunosuppressive molecules and soluble factors in the TME). Briefly, infused CAR-T cells need, in order to exert their cytotoxic effect, to: 1) traffic through the blood stream and migrate to the tumor tissue, in a chemokine directed manner, 2) cross the limiting blood vessels during the transmigration step 3) infiltrate the tumor and migrate to the vicinity of tumor cells by degrading TME components and 4) generate stable cell to cell contacts with tumor cells. Finally, success of adoptive cell therapy (ACT) is warranted by an increased persistence of infused CAR-T cells, which is dictated by their capacity to proliferate and survive in the hostile (acidic, hypoxic and nutrient and cytokine derived) tumor environment. Moreover, tumors have developed “escape mechanisms” in order to divert the immune-patrol process (21).

Therefore, CAR-T cell trafficking to and infiltration of the tumor is the first roadblock that needs to be overcome. Defective CAR-T cell infiltration is caused by: (i) chemokine/chemokine receptors mismatch or downregulated tumor-derived chemokines (22), (ii) an aberrant vasculature with downregulated or deficient adhesion molecules (23) and (iii) a remodeled tumor stroma, mainly composed of extracellular matrix (ECM) and cancer associated fibroblasts (CAFs) (24, 25).

#### 2.1.2 Overcoming the Mismatch or the Dysregulation of Chemokine Receptor/Ligand Axes

Recent studies have shown that endothelial cells lining the tumor vasculature are able to prevent the trafficking, the adhesion and to eventually hijack anti-tumor activity of T cells (26). Some tumors block T cell homing by reducing the expression of adhesion molecules such as ICAM-1, VCAM-1, and CD34 on the tumor endothelium (14). For instance, the overexpression of endothelin B receptors (ETBR) on the tumor vasculature in ovarian cancer represses T cell trafficking by preventing ICAM-1 clustering on endothelial cells, which has a central role in T cell arrest and migration (27). Furthermore, as CXCR3 and CCR5 are often used by activated T cell to infiltrate tumors that should express their respective ligands (28), an insufficient expression of CXCR3 and CCR5 ligands by some tumors leads to a decrease in T cell recruitment (29, 30).

**Figure 2:**
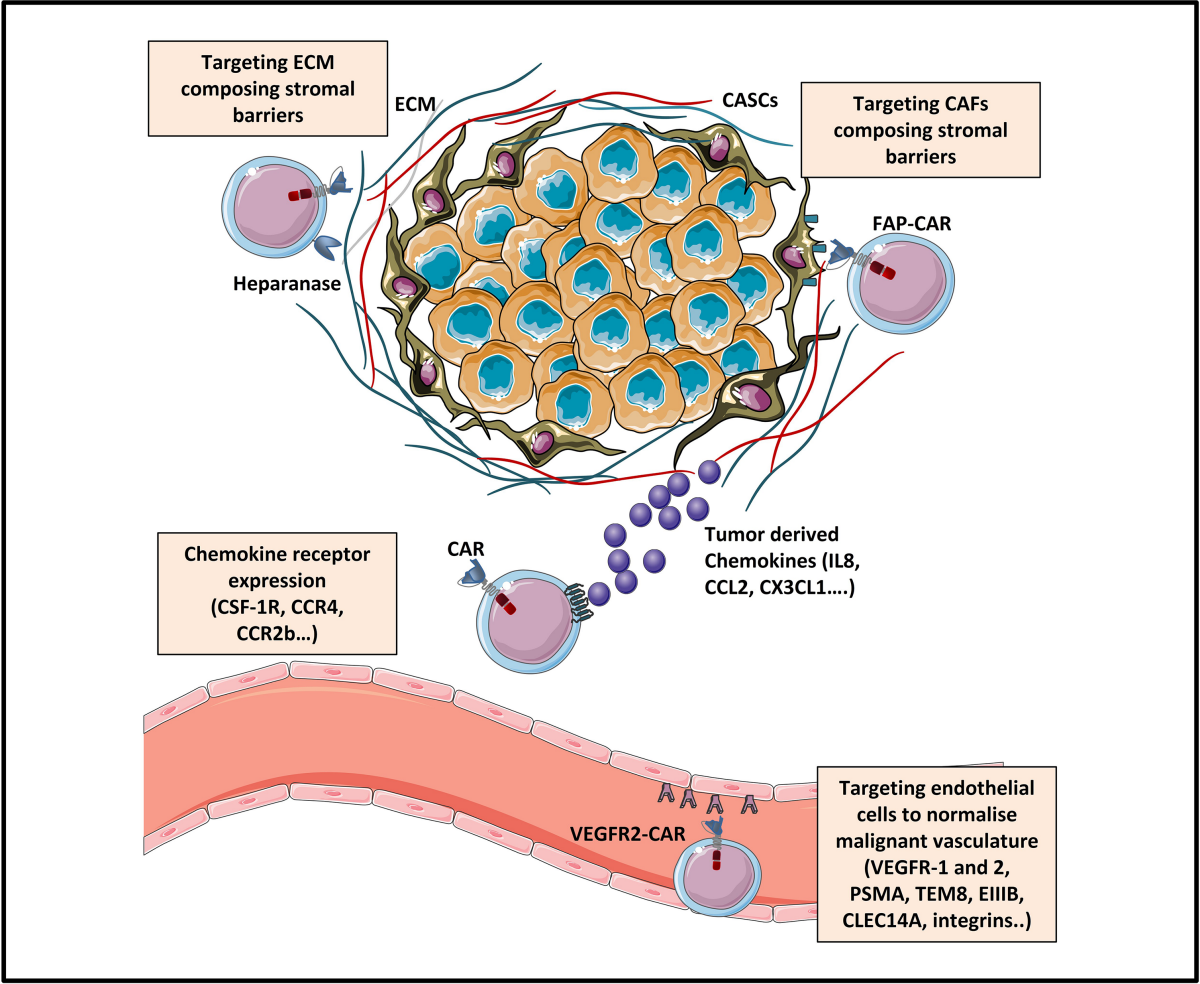
Strategies enhancing tumor trafficking and penetration [Adapted from Rafiq et al. (31)]. The trafficking of CAR-T cells towards tumor sites can be enhanced by engineering CAR-T cells expressing chemokine receptors (as for example CSF-1R, CCR4 or CCR2b) specific for tumor-derived chemokine ligands (IL8, CCL2, CXCL1…). Tumor penetration of CAR-T cells can be enhanced by various strategies: (1) normalizing the malignant vasculature by targeting tumor blood vessels *via* CAR targeting of endothelial/tumoral antigens (like VEGFR, EIIIB, TEM8, integrins.), and (2) targeting physical barriers in the tumor microenvironment (TME) like the extracellular matrix (ECM) or the cancer associated fibroblasts (CAFs).

Since efficient trafficking is the first critical step for CAR-T cells to mediate their anti-tumor activity, several strategies targeting chemokine‐chemokine receptor signaling are currently being explored in solid tumors. Some of them have already been tested in preclinical and clinical studies. To this end, CAR-T cells were genetically modified to co-express either chemokine receptors, among which we can cite: CCR2b, CXCR1/CXCR2, CCR4, CX3CL1, CSF-1R and CCR8 or to produce various chemokines: CCL19, CCL21 or CXCL11 (**Figure 2**). In more recent studies, co-expression of tissue homing molecules, as CD103 or CD39 was used to direct CAR-T cells to the tumor sites more efficiently

The chemokine ligand CCL2 or monocyte chemoattractant protein 1 (MCP-1) mediates the trafficking of immune cells into the TME in many types of malignancies, such as melanoma, colorectal, breast, prostate and pancreatic cancer (32). Therefore, co-expression of the CCL2 chemokine receptor, CCR2b, in CAR-T cells improves their anti-tumor activity, by enhancing their ability to traffic to the tumor bed. Craddock et al. demonstrated an improved homing (>10-fold) of GD2-specific CAR-T cells co-expressing CCR2b to CCL2-secreting neuroblastoma, as compared to CCR2-negative CAR-T cells (33). Likewise, co-expression of CCR2b was also associated with an increased migration (12.5-fold) of mesothelin (MSLN)-targeted CAR-T cells toward malignant pleural mesothelioma, in a study conducted by Moon et al. (34).

Furthermore, IL-8/CXCL8 was shown to be a pro-inflammatory chemokine that plays an important role in a variety of human cancers, including melanoma (35), prostate (36), colon (37), breast (38) and ovarian (39) cancers, by mediating tumorigenesis and angiogenesis. Some researchers took advantage of tumor-produced IL-8 in order to guide the IL-8 receptor (CXCR1 or CXCR2)-expressing CAR-T cells to infiltrate solid tumors (glioblastoma, hepatocellular carcinoma (HCC), ovarian and pancreatic cancer), and stimulate an antitumor immune response. Results showed a significantly enhanced tumor trafficking and persistence of genetically modified T cells, which triggered tumor regression, durable immunologic memory and better toxicity profile in mice (40–42). A clinical trial (NCT01740557) was initiated to evaluate the efficacy of T cells transduced with CXCR2 and with nerve growth factor receptor (NGFR), associated with Recombinant Human IL-2 (Aldesleukin) infusion in melanoma (**Table 10**). Exogenous supplementation of the Il2 vital support cytokine is widely used in the clinical setting (See *Targeting Fibroblast Activation Protein (FAP)* and *Tregs*), and is resumed in the table dedicated to CAR-T cells clinical trials (**Table 10**).

Moreover, it has been noted that two CCR4 ligands -CCL17 and CCL22-, are overexpressed in lymphoid malignancies such as Hodgkin’s lymphoma (HL) (43), and in many other types of human cancers (44) including ovarian (45), breast (46), esophageal (47) and gastric (48) cancers. The aberrant overexpression of those ligands at the tumor site plays a central role in recruiting CCR4+ Th2 and regulatory T cells (Tregs) to such malignancies, resulting in an immunosuppressive TME (43). Since CCR4- effector T cells are barely present at the tumor site, the forced co-expression of surface CCR4 in CAR-T cells appears to be a promising therapeutic strategy in the treatment of certain types of lymphomas. Taking advantage of a mouse model of HL, Di Stasi et al. demonstrated that CAR-T cells engineered to co-express the chemokine receptor CCR4 together with the effector antigen receptor CD30 (CAR-CD30 T cells), had improved migration towards the tumor and enhanced anti-lymphoma activity as compared to CD30 CAR-T cells lacking CCR4 expression (49). A clinical trial (NCT03602157) was initiated to ascertain the effectiveness of CAR-T cells co-expressing CD30 and CCR4 in relapsed/refractory CD30+ HL and cutaneous T cell lymphoma (CTCL) (**Table 10**).

More recently, mesothelin specific CAR-T cells (MSLN-CAR) transduced to express either CCR2b or CCR4 chemokine receptors of Mcp-1 were engineered by Wang et al. and tested *in vitro* and *in vivo* in a non-small-cell lung carcinoma (NSCLC) model. MSLN-CCR2b-CAR-T cells displayed superior anti-tumor function due to enhanced migration and infiltration into tumor tissues as well as no obvious toxicity (no organ damage). The MSLN-CCR4-CAR-T cells showed enhanced migration and potent cytotoxic function and cytokine production *in vitro* but were not further tested *in vivo* (50).

As previously mentioned, CXCR3 is highly expressed on effector T cells and plays a key role in their trafficking (51). Therefore, tumors expressing chemokines such as interferon-γ (IFN-γ)-inducible CXCR3 ligands would attract effector lymphocytes. CXCR3 binds three ligands: CXCL9 (monokine induced by IFN-γ), CXCL10 (interferon-induced protein-10) and CXCL11 (interferon-inducible T cell alpha chemoattractant) (52). Moon et al. used CAR-T cells as vehicles to deliver CXCL11 to the cancer site in order to increase its expression within the tumor and therefore recruit effector TILs. Unfortunately, this approach was not able to improve T cell tumor infiltration, despite of the local increase in CXCL11 (53). Given the success of oncolytic vaccinia viruses (VVs) expressing CXCL11 in increasing the numbers of effector T lymphocytes in specific murine tumors (54, 55), the same team combined the use of a VV engineered to produce CXCL11 with MSLN CAR-T cells administration. Results showed increased efficacy in CAR-T cells trafficking and tumor progression control of this combined strategy, as compared to VV.CXCL11 alone (53).

On another front, data showed that the unique member of the CX3-chemokine subfamily, termed fractalkine or CX3CL1, can be exploited to help overcome the poor homing of CAR-T cells to tumor sites. The CX3CL1-CX3CR1 axis is involved in chemotaxis and adhesion of leukocytes and in the recruitment of immune cell subpopulation such as NK cells, Th1 lymphocytes and macrophages (56). CX3CL1 is expressed in breast (57), pancreatic (58), gastric (59) and colon (60, 61) cancers. Siddiqui et al. demonstrated that CAR-T cells engineered to express CX3CR1 have increased infiltration towards CX3CL1-producing tumors in mice as well as decreased tumor growth (62).

In a proof-of-concept *in vitro* model, Lo et al. induced forced expression of the macrophage colony-stimulating factor 1 receptor (CSF-1R) to render CAR-T cells sensitive to CSF1, a monocyte recruiting chemokine enriched in various tumor tissues. Forced expression of CSF-1R exploits the T cell signaling machinery to enhance CAR-T cells Il-2 driven proliferation and costimulate production of IFN-γ, without reducing cytotoxicity and without inducing transdifferentiation to the monocytic/macrophagic lineage. CSF-1 forced expression is a cytokine engineering strategy which could improve both CAR-T cell effector function (i.e. persistence/proliferation and cytokine production) and CAR-T chemotaxis to the tumor site (63).

More recently, Cadilha et al. employed a combined CAR-T cells engineering strategy enabling enhanced recruitment by CCR8 expression together with shielding from immunosuppression by the expression of a dominant-negative TGF-β receptor 2 (TGF-β DNR). The team exploited the CCR8-CCL1 recruitment axis, by which various tumors with poor prognosis attract Tregs, to empower effector CAR-T cells with enhanced chemotaxis. The team validated this strategy in a murine model of pancreatic cancer and in human xenograft tumor models. Furthermore, this strategy exploits activated T cell derived CCL1 to potentialize a positive feedback loop in CCR8+ cells recruitment to the tumor site (64).

Two other teams designed fourth generation CAR-T cells producing/co-expressing both IL-7 and CCL19 or CCL21 (65, 66). These combinatorial strategies associating co-expression of chemokine receptors/ligands with production of homeostatic cytokines could enhance both migration of CAR-T cells to the tumor site and proliferation/persistence of CAR-T cells in the hostile TME. Adachi et al. engineered CAR-T cells specific to fluorescein isothiocyanate (FITC) co-expressing IL-7 and CCL19 (7 × 19 CAR-T cells), two factors produced by T-zone fibroblastic reticular cells and essential for the maintenance of T cell zones in lymphoid organs. Treated mice achieved complete remission of pre-established tumors and 7 × 19 CAR-T cells showed superior anti-tumor activity than conventional CAR-T cells, as well as an improved ability of both migration and proliferation in the TME. Response to 7 × 19 CAR-T cells was dependent on the recipient’s immune system (i.e activation and recruitment of dendritic cells and of tumor-reactive recipient T cells). Moreover, recipient conventional T cells also generated tumor –antigen-specific memory, probably due to epitope spreading. The authors raised security concerns about this engineering strategy, as gain of function (GOF) mutations of the IL-7 receptor (CD127) are frequent in pediatric T cell acute lymphoblastic leukemia (T-ALL) and as CCR7 could play a role in tumor metastasis. This engineering strategy could, therefore, benefit from the integration of a suicide gene system in order to prevent an eventual leukemic change of 7 × 19 CAR-T cells before clinical application (65). The same team validated the use of anti-mesothelin IL-7/CCL19-producing human CAR-T cells in a preclinical model of orthotopic pre-established malignant mesothelioma, as well as in patient derived xenograft (PTX) models of mesothelin-positive pancreatic cancer. As in the previous study, IL-7/CCL19-producing human CAR-T cells exerted a significant inhibition of tumor growth and prolonged survival of treated mice. Tumors showed increased infiltration with T recipient no-CAR-T cells as well as downregulation of exhaustion markers PD-1 and T cell immunoreceptor with Ig and ITIM domains (TIGIT) on T cells (67). Similar results were obtained with 7 × 19 CAR-T cells *in vivo* in the context of hepatocellular carcinoma (HCC) and pancreatic carcinoma (68).

There are two clinical trials on CAR-T cells co-expressing IL-7 and CCL19. Results from a first six-case cohort preliminary phase I clinical study (NCT03198546) in advanced HCC/PC/ovarian carcinoma (OC) patients with glypican-3 (GPC3) or MSLN expression have been published recently and show encouraging results: two complete responses (CR), two partial responses (PR) and 2 steady diseases (SD). There were no grade 2–4 adverse events or major complications (68). Another ongoing clinical trial (NCT03932565) evaluates intratumoral injection of Nectin4/FAP-targeted fourth-generation CAR-T cells (expressing IL-7 and CCL19, or IL12) for the treatment of Nectin4-positive advanced malignant solid tumors (NSCLC, breast, ovarian, bladder or pancreatic cancer). This represents an engineering strategy designed to enhance migration (CCL19), proliferation/maintenance (IL-7/IL-12) of CAR-T cells and to simultaneously target the stromal CAFs (anti-FAP). Three other clinical trials are evaluating the efficacy of this type of immunotherapy in the context of B cell lymphoma/multiple myeloma: NCT04833504 evaluating CD19-CAR-T expressing IL-7 and CCL19 in the context of relapsed/refractory B cell lymphoma, NCT04381741 evaluating CD19 CAR-T expressing IL-7 and CCL19 combined with PD-1 mAb for relapsed or refractory diffuse large B cell Lymphoma (DLBCL) and NCT03778346 evaluating fourth generation CAR-T cells simultaneously expressing IL-7 and CCL19 and directed against single or compound targets (Integrin β7, BCMA, CS1, CD38 and/or CD138) in the context of refractory/recurrent multiple myeloma (R/R MM). Results for the first two patients treated with BCMA-7 × 19 CAR-T cells (NCT03778346) in the context of R/R MM show encouraging results: an objective response within 1 month after BCMA-7 × 19 CAR-T cell infusion with one patient reaching CR and one a very good partial response (VGPR) and responses lasted more than 12-months (**Table 10**). There was no clinically significant toxicity. It is worth noticing that this CAR-T cell therapy was associated with a high proportion of stem cell memory (TSCM) among produced CAR-T cells, possibly due to IL-7 production (69). Indeed, several clinical studies have shown that the modifications to induce differentiation toward a TCM/TSCM profile improve CAR-T cell responses in subjects (70–72).

Another similar approach was to engineer Claudin18.2 (CLDN18.2)-specific CAR-T cells to co-express IL-7 together with the chemokine receptor CCR7 ligand CCL21 (7 × 21 CAR-T cells). CLDN18.2-specific second-generation CAR-T cells coexpressing IL-7 and CCL21 were tested *in vitro* and *in vivo* in three tumor models (breast, pancreatic and hepatocellular carcinoma) and revealed superior therapeutic effects to either conventional CAR-T cells or 7 × 19 CAR-T cells, without preconditioned lymphodepletion. As for 7 × 19 CAR-T cells, 7 × 21 CAR-T cells showed significantly improved survival and tumor infiltration. Treated mice showed increased infiltration of DCs as well as an inhibition of the tumor angiogenesis (presumed effect of CCL21) (66). No clinical trials evaluating the efficacy of CCL21 expressing CAR-T cells have been designed to date. However, various clinical trials use CCL21 gene modified dendritic cells (DCs-adenovirus CCL21) as anticancer vaccination strategies in lung cancer or melanoma (NCT00601094, NCT01433172, NCT01574222, NCT03546361 and NCT00798629).

Genetically engineered expression of TRM-type markers CD103 or CD39 on CAR-T cells has recently been evaluated as a strategy to overcome insufficient trafficking and infiltration of solid tumors (HCC) or hematologic cancers (human Raji lymphoma). In a HCC model, hepatitis B virus (HBV) surface protein-specific CAR-T cells (HBVsCAR-T cells) were genetically manipulated to express CD39 and showed increased cytotoxicity in an *in vitro* model of HCC organoids and T lymphocytes coculture and in a PDX mouse model. To prevent an exhausted phenotype of CD39+ CAR-T cells, the team used a combinatorial strategy of CD39 expression on CAR-T cells, together with knockdown of inhibitory immune-checkpoints (triple knockdown of PD-1, T cell immunoglobulin domain and mucin domain-3 (TIM-3), and lymphocyte-activation gene 3 (LAG-3) with shRNAs). CD39+ CAR-T cells showed enhanced cytokine production and antitumor effect. According to the authors, CD39 can serve as a biomarker to identify both personalized tumor-reactive CD8+ T cells as well as active CAR-T cells. Besides phenotypic identification, CD39 expression is also necessary for the cytotoxic effect of CD8 CARs and positively regulates antitumor activity (73). The TRM marker CD103 is a tissue homing molecule important for effector T cell trafficking as well as a promising prognosis biomarker for assessment of tumor-reactive TILS in various types of cancer, such as lung cancer, ovarian cancer and cervical cancers. CD103 is an integrin protein (αE) that binds integrin β7 to form the heterodimeric integrin complex αEβ7. Sun et al. used an E-Cadherin positive human lymphoma preclinical model (human Raji leukemia/lymphoma cells injected in NSG mice) to test therapeutic effects of CD103 expression on CD19-specific human CAR T cells. The gene encoding for the αE integrin was incorporated in the CD19-specific CAR structure to generate CD103-CD19-BBz-CAR T cells. These CAR-T cells showed more immature phenotypes (expressing high levels of CD62L and CD45RA), as compared to conventional CD19-BBz-CAR T cells, an increased production of IL-2 and greater expansion in culture, as well as improved anti-tumor efficacy (increased persistence, infiltration and eradication of lymphoma distant metastasis) upon adoptive transfer in immunodeficient mice (74).

The aforementioned preclinical studies on chemokine receptors expressing CAR-T cells are summarized in the table below (**Table 1**).

**Table 1:** Summary of preclinical studies on chemokine receptors/ligands or homing molecules expressing CAR-T cells.

Expressed chemokine receptor/ligand	CAR	Type of cancer	Reference
CCR2b	GD2	Neuroblastoma	(33)
	MSLN	Malignant pleural mesothelioma	(34, 50)
		Non-small-cell lung carcinoma (NSCLC)	
CXCR1 or CXCR2	CD70	Glioblastoma, ovarian or pancreatic cancer	(40)
	αvβ6	Ovarian or pancreatic cancer	(41)
	GPC3	Hepatocellular carcinoma	(42)
CCR4	CD30	HL	(49)
	MSLN	Non-small-cell lung carcinoma (NSCLC)	(50)
CCR8	MSLN	Pancreatic cancerPancreatic ductal adenocarcinoma (PDAC)	(64)
CXCL11	MSLN	Lung cancer	(53)
CX3CR1	CX3CR1	Colorectal cancer	(62)
CSF-1R	P28z	Prostate carcinoma	(63)
CCL19	FITC	MastocytomaLung carcinomaPancreatic adenocarcinoma	(65)
	MSLN	Malignant mesotheliomaPancreatic cancer	(67)
	GPC3	HCC	(68)
	MSLN	Pancreatic carcinoma	
CCL21	CLDN18.2	Breast cancerPancreatic carcinomaHCC	(66)
CD39	HBVs	HCC	(73)
CD103	CD19	Leukemia/Lymphoma	(74)

#### 2.1.3 Handling Neovasculature Aberrancies

Tumor angiogenesis is a hallmark of cancer growth and progression (75). The generation of a tumor-associated neovasculature enables the growing tumor mass to obtain nutriments and oxygen. Moreover, the tumor uses these new vessels as a principal route to enter the circulation and to metastasize and proliferate to distant areas (76). Tumor neovasculature is a disorganized labyrinth of vessels at risk of vascular collapse. It lacks a hierarchical vessel division, which gives rise to abnormal blood flow and permeability, diffusion-limited nutrient delivery, oxygen deprivation, and an increased interstitial fluid pressure in the tumor (77). Tumor-induced angiogenesis is induced by the imbalanced production of proangiogenic factors by the tumor cells, including vascular endothelial growth factor-A (VEGF), platelet-derived endothelial growth factor (PDGF), transforming growth factor (TGF)-α, angiopoietin (Ang), basic fibroblast growth factor (bFGF), fibroblast growth factor (FGF), and placental growth factor (PGF) (78, 79). These soluble factors bind to and activate diverse tyrosine kinase (TK) receptors, such as VEGFR1, VEGFR2, PDGFRA, and endothelial growth factor receptor (EGFR), promoting angiogenesis, among other biological events (80).

As stated previously, in order to reach the tumor site, T cells encounter a physical barrier, represented by this abnormal vasculature, which operates though as a first obstacle for lymphocyte recruitment into the tumor. Therefore, vascular targeting, using anti-angiogenic molecules, has been proposed as a novel strategy to block tumor growth. This approach aims at correcting the structural and functional abnormalities of the tumor vasculature, in order to improve T cell infiltration and immunotherapy efficacy (81). The first Food and Drug Administration (FDA) approval of an anti-angiogenic monoclonal antibody (mAb) (Bevacizumab) dates back to more than a decade ago (82). In more recent studies, substantial efforts were deployed to develop CAR-T cells with a chimeric receptor comprising a scFv antibody against specific angiogenic growth factors/receptors or adhesion molecules abnormally expressed on the tumor vasculature (83). To this end, Kershaw et al. were the first to suggest an indirect strategy to target stromal tumors by the usage of CAR-T cells targeting the vascular stroma instead of the cancer cell itself (84).

In order to inhibit tumor angiogenesis, Chinnasamy et al. genetically modified murine and human T cells to express a CAR targeted against VEGFR-2 (85). VEGFR-2 is overexpressed in tumor vasculature and is known to be critical for both physiological and pathological/tumor angiogenesis, as well as for VEGF-mediated tumor progression (86). VEGFR-2 is overexpressed in many types of solid tumors, including breast cancer, cervical cancer, NSCLC, hepatocellular carcinoma, and renal carcinoma (87). Chinnasamy et al. demonstrated that the antitumor effect of VEGFR-2 targeting CAR- T cells was not mediated through their direct cytotoxicity on the tumor cells but rather through their ability to eliminate VEGFR-2-expressing cells in the tumor vasculature. A single dose of VEGFR-2 CAR-T cells was effective in increasing tumor infiltration, and inhibiting the growth of 5 vascularized syngeneic tumors of various histological origins (85). The same group showed, in another study, that the coadministration of anti-VEGFR-2 CAR-T cells along with tumor-specific TCR transduced T cells (premelanosome (Pmel) TCR, tyrosinase-related-protein-1 (TRP-1) TCR, and tyrosinase-related-protein-2 (TRP2) TCR traduced T cells) resulted in a synergic anti-tumor effect and an extended tumor-free survival (TFS) of mice with metastatic melanoma tumors. These results emphasize the advantageous effects of dual targeting adoptive therapy including an anti-angiogenic strategy (88). Recently, Englisch et al. suggested that VEGFR-2 expressed on tumor vasculature could be a potential CAR target in Ewing sarcoma (EwS) (89), especially that this type of cancer is characterized by a limited TSA expression on cancer cells (90). Contact with their target triggered a powerful antigen‐specific degranulation response, increased proliferation and cytokine secretion of VEGFR-2 CAR-T cells. Data showed that VEGFR-2 CAR-T cells with short‐length or medium‐length hinge domains effectively destroyed VEGFR-2-expressing tumor‐associated endothelial cells (89). Similarly, in a study from Taheri et al. nanobody‐based anti-VEGFR2 CAR showed effective activation, degranulation and lysis of VEGFR2+ cell lines in an *in vitro* model (91). Unfortunately, adoptive transfer of VEGFR-2 CAR-T cells in a clinical setting was devoid of great success in a phase 1 clinical trial NCT01218867 on patients with metastatic cancer. The trial was terminated due to lack of objective responses: out of 24 infused patients, only one reached a PR and another one had a stable disease (SD) after CAR-T cell injection. There were no CR (**Table 10**).

While VEGFR-2 plays a critical role both in physiological and pathological angiogenesis, VEGFR-1, another member of the VEGFR family, is strictly involved in pathological angiogenesis (92). Even though both are abnormally expressed at high levels on tumor vasculature, their signaling characteristics are different (93). However, VEGFR-1 is not restricted to endothelial cells as expression has also been proven on monocyte/macrophages, and on various types of tumor cells (92). VEGFR-1 has been shown to be a key regulator of macrophage’ function and of cancer metastasis, among others, which makes it an interesting target in the development of novel approaches for cancer ACT (94). Wang et al. demonstrated that VEGFR-1 CAR-T cells can be a promising solution to break the resistance to traditional anti-angiogenic therapies, with higher efficacy than strategies blocking separately cancer growth or angiogenesis. This study also showed that co-administration of IL-5 producing CAR-T cells enhanced the anti-metastasis activity mediated by VEGFR-1 CAR-T cells (95).

Prostate-specific membrane antigen (PSMA) is another transmembrane protein highly expressed on the tumor-associated endothelium of a great variety of solid tumors - including bladder, oral, hepatocellular, gastric, colorectal, breast, ovarian, renal, and pancreatic ductal carcinoma as well as NSCLC and melanoma - (96, 97). Although not expressed by the normal endothelium, like it is the case of VEGFR, PSMA is still expressed at low levels in normal tissues as the brain, liver, kidney, intestine, colon and the prostate (98). Moreover, PSMA has a crucial role in tumor neovascularization. Santoro et al. directed a proof-of-concept study showing that PSMA CAR-T cells can recognize primary tumor PSMA-expressing endothelial cells and disrupt the tumor vasculature both *in vitro* and *in vivo*. Contrary to traditional anti-angiogenic agents, anti-PSMA CAR-T cells showed long-term *in vivo* persistence. However, in order to improve the safety profile of PSMA CAR-T cells, toxicity control mechanisms like the use of split-signaling CAR-T cells should be needed (97). PSMA has especially been targeted in prostate cancer patients, with various ongoing clinical trials (NCT01140373 (99, 100), NCT01929239, NCT00664196; and NCT03089203) (*see Tregs* and **Table 10**) (101).

Tumor endothelial marker 8 (TEM8), also known as anthrax receptor 1 (ANTRX1), is another cell membrane glycoprotein consistently overexpressed in the tumor vasculature and in many types of cancer, including breast (102), gastric (103),, skin (104), colon (105), and lung (106) cancers. Blocking or knocking out TEM8 inhibited pathological angiogenesis in several preclinical cancer models (104, 107). Moreover, anti-TEM8 CAR-T cells can serve as a potential targeted therapy for triple-negative breast cancers (TNBC). Results from Byrd et al. showed that TEM8-targeted CAR-T cells were able to concomitantly destroy TNBC tumor cells, breast cancer stem-like cells (BCSC) as well as tumor endothelial cells, and to cause regression of lung metastatic TNBC cell line-derived xenograft tumors (108). Unfortunately, a study published by Petrovic et al. raised concerns over possible on-target/off-tumor toxicities of TEM8-specific CAR-T cells (109).

It has been shown that fibronectin (FN) splice variants EIIIA and EIIIB are overexpressed in the vasculature of many types of tumors, including breast, lung and prostate cancers and high-grade glioma, whereas absent in normal tissues (110–112). These properties make EIIIA and EIIIB ideal targets for CAR-T cell therapy. Genetically engineered CAR-T cells targeting EIIIB were able to inhibit the growth of solid cancers in immunocompetent mice by compromising the blood supply of the tumor (113). Based on three tumor models, Wagner et al. reported similar results using immunodeficient mice treated with anti-EIIIB CAR-T cells (114).

Recently, C-type lectin domain family 14 member A (CLEC14A) has been identified as part of a molecular gene signature for tumor angiogenesis based on a meta-analysis on breast cancer, head and neck squamous cell carcinoma (HNSCC), and clear-cell renal cell carcinoma (ccRCC) (115). This protein is mainly overexpressed in the three aforementioned cancers (116, 117). CLEC14A could be a promising target for antiangiogenic therapy. A single injection of CLEC14A-specific CAR-T cells was sufficient for a significant suppression of tumor growth in 3 distinct tumor models. Use of anti-CLEC14A CAR-T cells could be combined with CAR-T cells targeting another tumor endothelial marker, in order to increase tumor vessel targeting capacities (118).

The integrin αvβ3 emerges as another potential target for cancer immunotherapy. Integrin αvβ3 is expressed on different types of cancer, including glioblastoma (119), melanoma (120), pancreatic (121), breast (122) and prostate cancers (123). Even though expressed on activated endothelial cells and newly formed vessels, it is not detectable in resting endothelial cells and normal tissues, making it a valid target for the treatment of many solid tumors (124). Wallstabe et al. generated αvβ3 targeted CAR-T cells and investigated antitumor effects of such approach in preclinical models *in vitro* and *in vivo*. They concluded that this strategy was able to inhibit tumor growth, but without achieving tumor eradication. Presence of haematomas in the tumor tissues proved that engineered T cells damaged tumor vessels, due to αvβ3-expression on tumor endothelium. Results also showed that adoptive therapy with αvβ3 CAR-T cells was more effective than immunotherapy with anti-αvβ3 mAbs (125).

Another integrin, the integrin αvβ6, whose expression on endothelial cells is restricted to development and remodeling processes (like wound healing, chronic inflammation and cancer), is upregulated in various cancers (126) and associated with more invasive tumor phenotypes, characterized by high tumor invasion and shorten survival in colon and cervix cancers or in NSCLC (127). This integrin emerged as an interesting target for immunotherapy with CAR-T cells in cholangiocarcinoma (CCA), a lethal bile duct cancer with poor responses to classic therapy. Targeting of CCA with anti- αvβ6 fourth generation CAR-T cells showed anti-tumor function against αvβ6 expressing CCA tumor spheroids, *in vitro* (128). In a previous study from Whilding et al., anti-αvβ6 CAR-T cells showed *in vivo* efficacy in other solid tumors expressing intermediate to high levels of this integrin (ovarian, breast, and pancreatic tumor xenografts in SCID beige mice). For selective expansion, CAR-T cells were engineered to co-express the IL-4-responsive fusion gene (4αβ, obtained by fusing the human IL-4 receptor α ectodomain to the shared human IL-2/IL-15 receptor β transmembrane and endodomain regions). Moreover, despite expression of this integrin in non-tumor endothelium, toxicities related to anti-αvβ6 CAR-T infusion were mild and reversible and only associated to systemic infusion of supra-therapeutic doses (129). There is no ongoing clinical trial with anti-αvβ6 CAR-T cells in cancer patients, but anti-αvβ6 cancer targeting, either by monoclonal antibodies or by peptides has already been tested in *in vitro* or preclinical animal models of breast (130) and pancreatic cancers (131, 132).

The aforementioned preclinical studies on proangiogenic factors/receptors-targeting CAR-T cells are summarized in the table below (**Table 2**).

**Table 2:** Summary of preclinical studies on proangiogenic factors/receptors-targeting CAR-T cells.

Target	Type of cancer	Reference
VEGFR-2	Solid tumors	(85)
	Metastatic melanoma	(88)
	EwS	(89)
	Experimental cancer	(91)
VEGFR-1	Lung cancer	(95)
PSMA	Ovarian cancer	(97)
TEM8	TNBC	(108)
EIIIB	Solid tumors	(113)
	Lung cancerSarcomaHigh-grade glioma	(114)
CLEC14A	Lung carcinomaPancreatic cancer	(118)
αvβ3 integrin	Metastatic melanoma	(125)
αvβ6 integrin	Cholangiocarcinoma(CCA)	(128)
	Ovarian, breast and pancreatic cancer	(129)

#### 2.1.4 Targeting the Tumor Stroma

Besides strategies aiming at targeting tumor blood vessels, engineering modifications targeting stromal cells may also be promising strategies for CAR-based immunotherapy. Targeting non-cancer cell components of the tumor stroma could help to enhance the anti-cancer effect of this immunotherapy for many reasons. First, stromal cells are less prone to immune-escape from the CAR-T cells attack as they show higher genetic stability than tumor cells, and are less likely to lose antigen expression *via* immunoediting (133). Second, since tumor stroma can be found in almost all human adenocarcinomas, CAR-T cells targeting the extracellular matrix (ECM) and/or the nonmalignant cancer-associated stromal cells (CASCs) could generate “broad-spectrum” CAR-T cells (134). Finally, tumor stroma plays a major role in tumor survival, growth, invasion, and angiogenesis, by producing growth factors, and chemotactic factors that attract immunosuppressive cells, and by expressing inhibitory surface checkpoint proteins (135). However, as extracellular matrix components are vital components of connective tissues, this targeting strategy needs identification and usage of specific tumor-ECM targets, in order to avoid on-target/off-tumor toxicities. To this regard, some studies focused on targeting ECM components by using CAR-T cells expressing ECM degrading enzymes while others chose as an attractive stromal candidate the fibroblast activation protein (FAP) expressed in CASCs (**Figure 2**).

a. ECM components modifying enzymes

Another strategy aiming at facilitating cellular penetration into solid tumors is genetic manipulation of CAR-T cells to secrete ECM-modifying enzymes. Indeed, the ECM is a complex structural component of the TME and the main physical barrier that hinders T cell-cancer cell contacts. The ECM is synthesized by malignant cells and cancer associated fibroblasts (CAFs) and can constitute up to 60/% of the tumor mass (136). Different ECM molecules, such as fibrillar collagen, hyaluronan (HA), proteoglycans (chondroitin sulfate, dermatan sulfate, heparan sulfate, and keratan sulfate), elastin, fibronectin and laminins are highly expressed in many solid cancers (136). Therefore, in order to access the tumor sites and mediate their anti-tumor functions, T cells must be able to degrade the main components of the ECM. Lymphocytes secrete specific enzymes to disrupt the ECM, including: (i) heparinase (HPSE), an endoglucuronidase that cleaves heparan sulfate side chains of heparan sulfate proteoglycans (137), (ii) hyaluronidase, an endoglycosidase that cleaves glycosidic bonds of hyaluronic acid (138), and (iii) matrix metalloproteinases (MMPs), endopeptidase proteases that cleave the majority of ECM and non-ECM components (**Figure 2**). Caruana et al. noted that *in vitro*-engineered and cultured T cells lose heparinase expression following *TP53* binding to the HPSE gene promoter, which may restrict CAR-T cell infiltration in stroma-rich solid tumors. To this regard, the authors engineered CAR-T cells to express heparinase and demonstrated that it’s expression led to improved cell migration in neuroblastoma xenograft models (139). Xiong et al. studied, *in vitro* and *in vivo*, the ability of GPC3 (Glypican 3 protein)-targeted CAR-T cells co-expressing IL-7 and the PH20 hyaluronidase to infiltrate hepatocellular carcinoma (HCC) xenograft models. Their results showed that the co-expression of the two aforementioned genes improved CAR-T cells trafficking, which may significantly enhance their efficacy in solid tumors (140).

Similarly, Zhao et al. reported the construction of MSLN (mesothelin)-targeted CAR-T cells with the overexpression of a secreted form of the human hyaluronidase (sPH20-IgG2) and found that this enzyme can promote the antitumor activity of these CAR-T cells *in vitro* and *in vivo* in gastric cancer cell xenografts, by promoting their infiltration (141). Use of a pegylated form of the human recombinant hyaluronidase (PEGPH20) has already been tested in the clinical setting in two randomized trials, as an adjuvant to chemotherapy in metastatic pancreatic adenocarcinoma (142, 143). Results of one of the trials (NCT01959139) could claim certain caution as adjuvant PEGPH20 therapy resulted in a diminished OS as well as an increased toxicity (gastrointestinal and thromboembolic events) (142). The other clinical trial (NCT01839487) did not confirm the reduction in survival (143) (**Table 10**). Even so, both studies confirmed an increased thromboembolic risk of the PEGPH20 therapy and imposed the adjunction of an anticoagulant prophylaxis with low molecular weight heparin in the study from Hingorani et al. (143). In a more recent phase III trial adjunction of PEGPH20 to chemotherapy had no benefits in terms of OS or progression free survival (PFS) in the case of metastatic pancreatic carcinoma (144).

Not least, another strategy to enhance CAR-T cells migration through the collagen barriers of the ECM could be CAR-T cell production of another ECM-modifying enzyme, the MMP8 metalloproteinases (also known as collagenase-2), as suggested by Mardomi and Abediankenari (145). However, transgenic production of MMPs has not been applied yet to CAR-T cells engineering. This type of engineering strategy, can also seam tempting for genetically engineered Macrophages (CAR-Macrophages) (146).

The aforementioned preclinical studies on CAR-T cells expressing ECM degrading enzymes are summarized in the table below (**Table 3**).

**Table 3:** Summary of preclinical studies on CAR-T expressing ECM degrading enzymes.

ECM degrading enzyme	CAR	Type of cancer	Reference
HPSE	GD2	Neuroblastoma	(139)
PH20	GPC3	HCC	(140)
	MSLN	Gastric cancer	(141)

b. Targeting Fibroblast Activation Protein (FAP)

**Figure 3:**
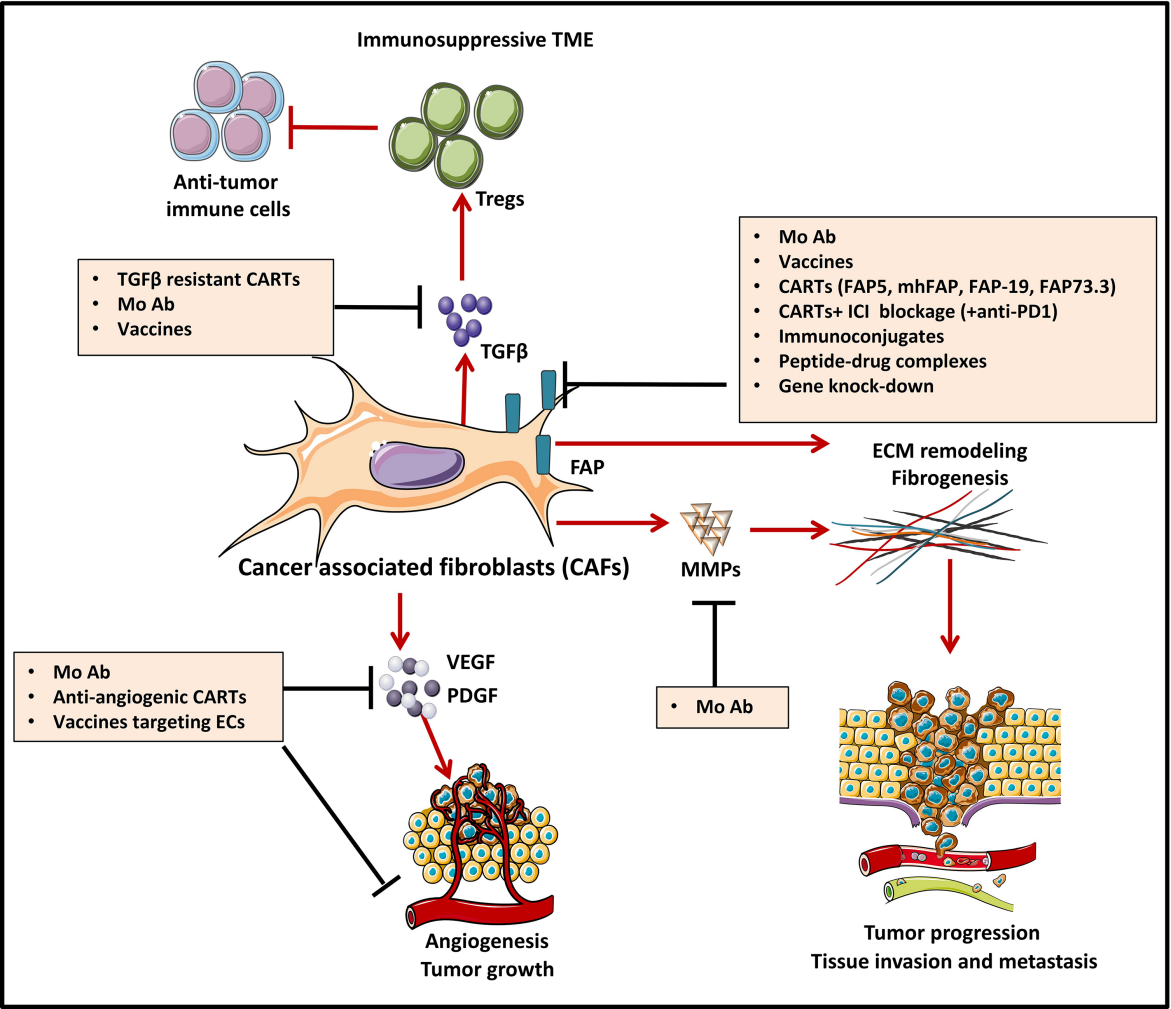
Strategies to counteract protumorigenic effects of CAFs [Adapted from Kakarla et al. (148)]. Cancer associated fibroblasts (CAF)-directed anti-cancer therapies are one of the weapons of tumor targeting which is directed against the stromal compartment. Strategies depicted in this figure aim at inhibiting cancer associated fibroblasts (CAFs) functions and are based on targeting crucial signals and effectors of CAFs such as cytokines (TGFβ) and growth factor pathways (VEGF, PDGF…). For instance, CAF-derived extracellular matrix proteins (MMPs) and associated signaling can be targeted with monoclonal antibodies (MAb), to induce stromal depletion and increase immune T cell infiltration. Blocking some targets like TGFβ, can act both upstream and downstream, by blocking CAF formation and attenuating downstream signaling in CAFs that are already established. FAP targeting aims at blocking CAFs ability to exert tumor promoting effects in the TME. Targeting FAP can be done by using either MAb/antibody-drug conjugates, immunoconjugates or peptide-drug complexes, FAP-specific CAR-T cells or strategies of gene-knock out. Some other strategies, not depicted in this figure aim at CAFs direct depletion or CAFs normalization towards an inactive phenotype.

Growing evidence proves that many cell types within the TME play a key role in oncogenesis. Among them, CAFs, a major component of the tumor stroma, represent a reactive tumor-associated fibroblast population that secretes various active factors promoting tumor development, progression, metastasis, and therapeutic resistance (147) (**Figure 3**). CAFs express various molecules that can be targeted by immunotherapies. Among them, FAP has recently emerged as the most promising target (149). FAP is a cell surface serine protease that is highly expressed on the CASCs of various human cancer types (150), such as lung (151), prostate (152), pancreatic (153), colorectal (154), and ovarian cancer (155). In contrast, the expression of this proteolytic enzyme on normal quiescent adult stromal cells and benign tumors is reported to be low to undetectable. Moreover, several studies have shown that tumors expressing FAP are associated with poor prognosis (150), enhanced tumorigenesis (150) and an increased neo-angiogenesis (156). Therefore, different strategies have been used to target FAP using antibodies (157, 158), vaccines (159, 160), immunoconjugates (161, 162), peptide-drug complexes (163–166), FAP gene knock-down by siRNA delivery (167), and CAR-T cells (168).

A large number of preclinical studies using FAP-targeted CAR mouse T cells have been reported to date (**Table 4**). Tran et al. genetically modified T cells to express a scFv from the FAP-specific monoclonal antibody (MAb) FAP5, reactive both to human and mouse FAP. They report effective cytotoxic effect of FAP-reactive CAR T cells *in vitro*. However, adoptive transfer of FAP5-CAR-T cells into mice bearing a variety of subcutaneous tumors mediated limited antitumor effects and induced significant cachexia and lethal bone toxicities in two mouse strains, due to low-level expression of FAP in multipotent bone marrow stromal cells (BMSCs) (169). Moreover, low level expression of FAP has been documented in other healthy tissues like: adipose tissue, skin, muscle and pancreas (150). Other on-target/off tumor toxicities after FAP+ stromal cell depletion with CAR-T cells, reported by Roberts et al., were bone marrow hypoplasia, anemia, pancreatic toxicity and loss of muscle mass (175). In an established lung cancer model, Kakarla et al. generated a CAR specific for both murine and human FAP (mhFAP) using the scFv from MO36 (previously generated by phage display from an immunized FAP/knock-out mouse) (176). They noted that mhFAP CAR-T cells were able to significantly reduce FAP+ stromal cells and tumor growth, with no toxicity or negative effects on wound healing. This study shed the light on the advantage of co-targeting CAFs and cancer cells since the authors demonstrated that combining mhFAP CAR-T cells with EphA2 CAR-T cells increased overall antitumor activity (170). Schuberth et al. developed FAP CAR-T cells using F19 CAR that only recognizes the human version of FAP. They removed the binding site of lck from the CD28 intracellular signaling domain in order to impede IL-2 secretion upon FAP CAR-T cell engagement with its target, and thus reduce Tregs persistence. The authors found that the redirected T cells successfully lysed FAP+ mesothelioma cells in an antigen-specific manner *in vitro* and *in vivo*. However, the authors could not evaluated the on-target/off-tumor toxicity of their CAR-T cells since F19-FAP antibody targets only the human version of FAP, with no cross-reactivity with the mouse version (171).

**Table 4:** Summary of preclinical studies on FAP-targeted CAR-T cells.

CAR	Intracellular signaling domains	T cell origin	Type of cancer	Reference
FAP-5	CD28, 4-1BB and CD3ζ	Mouse	MelanomaColorectal cancerPancreatic cancerBreast cancer	(169)
mhFAP	CD28 and CD3ζ	Human	NSCLC	(170)
FAP-F19	ΔCD28 and CD3ζ	Human	Mesothelioma	(171)
FAP-73.3	CD8α, 4-1BB and CD3ζ	Mouse	MesotheliomaLung cancer	(172)
FAP-73.3	KIR2DS2 and DAP12	Human	Mesothelioma	(173)
FAP-F19 (+ Anti-PD-1)	ΔCD28 and CD3ζ	Human	Mesothelioma	(174)

Wang et al. developed FAP-73.3 CAR mouse T cells against mouse FAP and demonstrated that depletion of FAP+ cells reduced tumor growth in an immune-dependent manner, as the antitumor effect was only seen in fully immunocompetent mice. Moreover, no clinical toxicities have been observed in mice following the administration of FAP-73.3 CAR mouse T cells *in vivo*. In order to enhance the antitumor activity, the authors successfully increased the efficacy of their FAP CAR-T cells either by reinfusing a second dose one week later or by combining the redirected T cells with an HPV-E7 vaccine (Ad.E7) (172). The same group designed an alternative chimeric immunoreceptor by fusing the FAP CAR to the transmembrane and cytoplasmic domains of KIR2DS2, a stimulatory killer immunoglobulin-like receptor (KIR), instead of the conventional cytoplasmic domain of CD28 used previously. The aim of this study was to evaluate whether KIR-based CAR-T cells expressing FAP-KIR2DS2 and DAP12 (an immunoreceptor tyrosine-based activation motif (ITAM)-bearing transmembrane adaptor associated with NK-activating receptors) can exhibit a more powerful antitumor response as compared to CD3ζ-based CAR-T cells. Therefore, they generated murine FAP-KIRS2/DAP12-modified T cells using the same scFv from the FAP-73.3 hybridoma. Results showed an enhanced antitumor effect with a complete inhibition of tumor growth, as compared to the significant but minimal slowing of tumor growth with CD3ζ-based CAR-T cells. However, despite the lack of toxicity of the CD3ζ-based FAP-specific CAR-T cells, FAP-KIRS2/DAP12 CAR-T cells showed similar toxicity to the one reported by Roberts et al. in the aforementioned study, suggesting that higher efficacy of FAP targeting is also associated with higher risk of on-target/off-tumor toxicity (173). This issue prompted Gulati et al. to investigate which intracellular signaling domains should be combined with FAP CAR for malignant pleural mesothelioma treatment. When comparing CAR-T cells expressing the CD28/CD3ζ, ΔCD28/CD3ζ and 4-1BB/CD3ζ CAR, the authors noted that 4-1BB/CD3ζ CAR-T cells persisted the most (until day 44) in the peripheral blood of humanized mice, and that the deletion of lck in ΔCD28/CD3ζ CAR enhanced antigen-specific proliferation. Despite higher persistence of 4-1BB/CD3ζ CAR-T cells, statistically significant tumor control *in vivo* was only obtained when combining FAP-ΔCD28/CD3ζ CAR-T cells with the immune checkpoint PD-1 inhibitor antibodies (174).

To date, two clinical trials using FAP CAR-T cells have already been conducted. The first one is a phase I clinical trial (NCT01722149) using CD3ζ/CD28-based FAP-specific CAR-T cells in three patients with malignant pleural mesothelioma (**Table 10**). A single dose of 1x 10^6^ CAR-T cells was administered through a pleural catheter. This therapy was well tolerated without any significant toxicity. In addition, one of the three patients received an anti-PD-1 checkpoint inhibitor antibody 8 months after FAP CAR-T cell administration; no clinical toxicity has been reported and 2 out of 3 patients were still alive after a follow-up of 18 months (177, 178).

The second one, cited earlier, is a phase I clinical trial (NCT03932565) using fourth-generation CAR-T cells coproducing IL-7 and CCL19/IL-2 in patients with Nectin4-positive advanced malignant solid tumors such as NSCLC, breast, bladder, pancreatic and ovarian cancer. An approach of intravenous infusion combined with intratumoral injection of Nectin4/FAP-targeted CAR-T cells will be undertaken. The clinical trial is ongoing and still recruiting (**Table 10**).

### 2.2 Counteracting the Immunosuppressive TME

**Figure 4:**
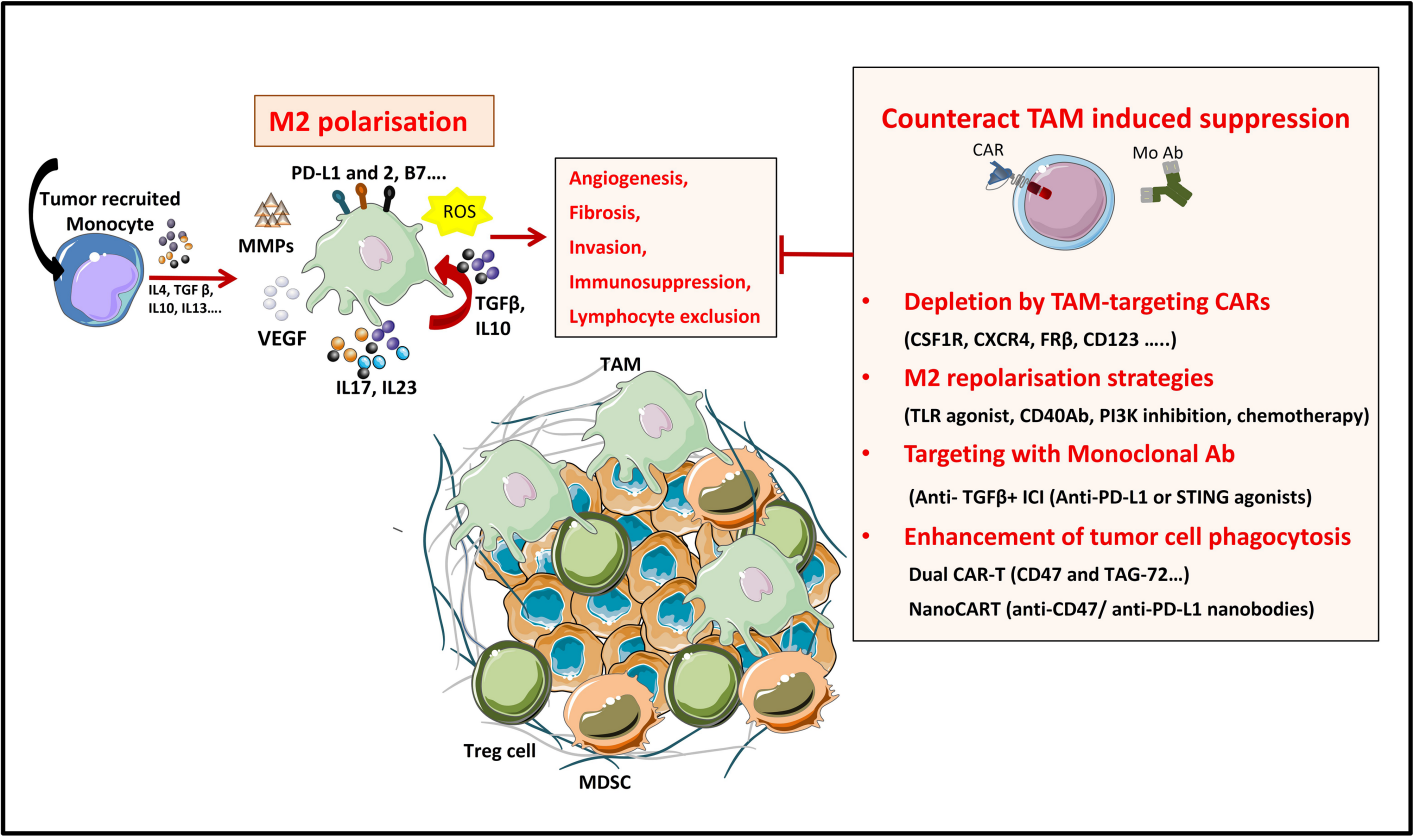
Strategies to overcome TAM’s induced suppression in the TME. TAMs are a tumor promoting immune populations derived under a specific cytokine milieu either from blood circulating monocytes or from tumor resident macrophages. TAMs exert their tumor promoting and immunosuppressive role by means of cell-to cell contact (inhibitory check point ligands), by secreting soluble factors (like cytokines IL10, IL17, L23), by producing ECM-modifying enzymes (MMPs) or by producing reactive species of oxygen (ROS). All these factors promote tumor progression. TAMs directed therapies in the TME aim either at (1) specifically depleting the TAM population, at (2) reprogramming M2 towards proinflammatory M1 phenotypes, at (3) targeting TAM-secreted factors or 4) at enhancing TAM’s phagocytic functions in the TME.

The solid tumor microenvironment is composed, as stated previously, by stromal cells (including CAFs), surrounded by the tumor vasculature and by an immune infiltrate of immunosuppressive cells, among which myeloid cells (myeloid-derived suppressor cells or MDSCs), tumor-associated macrophages (TAMs) and Tregs (**Figure 4**). As previously shown (see *Targeting the Tumor Stroma*), stromal cells strongly impact the TME as well as the interactions between the immune system and the tumor. Various cell types of hematopoietic origin contribute to the generation of an immunosuppressive TME. This immunosuppressive TME is maintained both by contact mechanisms as cancer cells and stromal cells express a broad range of inhibitory immune-checkpoint ligands (for PD-1, TIGIT, LAG-3 and TIM-3) and by suppressive soluble factors produced by immune cells or by CAFs (cytokines like TGF-β or IL-10). Moreover, other soluble factors with known effects on angiogenesis and produced by this pro-tumorigenic cells, like VEGFA and prostaglandin E2 (PGE2), also induce immunosuppression by inhibiting cytotoxic T lymphocytes (CTLs) and NK cells and by inducing accumulation and proliferation of Tregs (179–181). Therefore, targeting immunosuppressive cells in the TME could improve the efficacy of immunotherapies by increasing tumor recognition by the immune system. In this section, we will discuss the mechanisms by which pro-tumorigenic immune cells from the TME hijack T cell function as well as the different molecular strategies deployed to enhance the efficacy of genetically modified T cells to surmount these roadblocks.

#### 2.2.1 TAMs

Macrophages, one of the main effector cells of the immune system, play a key role in both innate and adaptive immune responses. They constitute the first line of defense against foreign pathogens and help trigger an adaptive antigen-specific response. Macrophages are potent immune effector cells with extensive plasticity and heterogeneity. Some types of macrophages play a crucial role in maintaining tissue homeostasis, by promoting wound healing, whereas others promote inflammation (182). Moreover, impaired macrophage function may lead to the development of many pathologies such as cancer (183). Macrophages are polarized into two contrasting groups: classically activated macrophages or M1 macrophages (pro-inflammatory and usually anti-tumor) and alternatively activated macrophages or M2 macrophages (anti-inflammatory and pro-tumor). This polarization is induced by exposure to soluble factors or pathogen derived molecules in the tissues. M1 macrophage polarization is driven by GMCSF, IFN-γ, TNF-α, lipopolysaccharide (LPS), or other pathogen-associated molecular patterns (PAMPs). M1 macrophages are proinflammatory and play an important role in anti-tumor immunity by: (i) orienting cellular immunity towards à TH1 type response by secreting TNFα, IL-1β, and IL-12, (ii) recruiting Th1 lymphocytes to sites of inflammation through secretion of CXCL9 and CXCL10 chemokines and (iii) presenting processed antigens and expressing costimulatory molecules which enhance T cell responses (184). M2 polarization on the other hand, occurs in the presence of cytokines like MCSF, IL-4, IL-10, IL-13, or TGF-β. Despite their role in tissue homeostasis (stimulating Th2 responses to eliminate parasites, immune regulation, wound healing and tissue repair), M2 macrophages can also promote tumor progression.

Tumors secrete and produce a variety of soluble and mechanical factors to recruit both circulating monocytes and tissue resident macrophages to the TME and convert them to TAMs. TAMs are a specialized population of M2-like macrophages, located in the TME, that share some phenotypic characteristics with M1 and M2 macrophages but have a particular transcriptional profile which is distinct from both types. TAMs enhance tumor progression and metastasis by promoting genetic instability and by enhancing angiogenesis, fibrosis, invasion, immunosuppression and lymphocyte exclusion (185, 186).

On the one hand, TAMs produce inflammatory cytokines like IL-17 and IL-23, which increase genetic instability and on the other hand they can impede tumor immunosurveillance, and thus T cell-mediated antitumor immunity, by secreting immunosuppressive cytokines like TGF-β and IL-10, by expressing immune checkpoint ligands such as PD-L1, PD-L2, B7-H4, or VISTA (4, 187) or by producing reactive oxygen species (ROS). Furthermore, immunosuppressive cytokines produced by TAMs have a role in Treg recruitment. Nonetheless, other factors produced by TAMs are VEGF and MMP enzymes, which promote tumor angiogenesis and metastasis by inducing TME remodeling, increased blood vessel formation, and tumor cell migration (184). All these characteristics make TAMs targeting a promising strategy for cancer treatment (188).

Up to date, various therapeutic strategies targeting TAMs have already been tested in preclinical studies and clinical trials (**Figure 4** and **Table 10**) (189). Macrophage-focused immunotherapeutic strategies aimed either to deplete or to repolarize TAMs. Therefore, the first approach was to reduce or deplete TAMs by eliminating existent TAMs or by inhibiting further TAM recruitment, by targeting: (i) colony-stimulating factor 1 (CSF1)/CSF1 receptor (CSF1R) signaling pathway (190), (ii) chemokines/chemokines receptors axis such as CCL2/CCR2, CCL5/CCR5 (191, 192), (iii) IL-8/CXCR2 (193) or (iv) CXCL12/CXCR4 axis (194).

Second approach is to repolarize TAMs toward an M1-like phenotype, by inhibiting the PI3Kγ signaling pathway (195), by triggering inflammatory activating toll-like receptor (TLR: TLR3, TLR4, TLR7/8 and TLR9) signaling pathway with TLR agonists (196), or by using agonistic CD40 antibodies (197). Third approach of TAM reprogramming is to promote antigen presentation and phagocytosis of TAMs by blocking anti-phagocytic surface proteins called “don’t eat me” signals, like SIRPα or Siglec-10, with antibodies blocking CD47 or CD24 expressed on cancer cells (198, 199) (**Figure 4**).

Nonetheless, another molecule of interest for targeting TAMs is TGF-β, an anti-inflammatory cytokine typically expressed by macrophages during injury resolution. Macrophages are both a source and a target for TGF-β, causing a positive feedback loop for TAMs and maintaining the immunosuppressive TME by promoting the secretion of additional TGF-β. TAM targeting by TGF-β blockade has already been employed, either in association with STING agonists or with anti-PDL1 blockade and showed tumor regression in preclinical models (200–202). For example, STING agonists DMXAA and cGAMP promote CAR-T cell persistence in the TME of immunocompetent mice in a breast cancer preclinical model. Association of STING agonists with CAR-T cell immunotherapy reprograms macrophagic and myeloid immunosuppressive populations in the TME. This is proven by an increased expression of CXCL9 and CXCL10 by myeloid cells within the TME, with increased recruitment of CXCR3+ TH1 to the tumor as well as by the enhanced expression of genes associated with M1-like macrophages and a marked loss of genes associated with M2-like macrophages and MDSC-like cells (201).

Additional potential molecular targets are discussed by Li et al. in a recent review (189). An increased research aimed at identifying TAM-associated or even TAM-specific targets and some have been used to redirect CAR-T cells against TAMs. Lynn et al. identified, in 2015, folate receptor beta (FRβ), a glycophosphatidylinositol-anchored receptor, as a potential target, as it is highly expressed in monocyte-derived TAMs from primary ovarian cancer. They, thereby, developed mouse FRβ-specific CAR-T cells to target the immunosuppressive M2-like subset of TAMs, while sparing M1-like subpopulations. The preliminary data showed that adoptive transfer of FRβ CAR-T cells into ID8 tumor-bearing mice depleted FRβ+ TAMs and delayed tumor development (203). Similarly, in the study from Rodriguez-Garcia et al., infusion of FRβ-specific CAR-T cells resulted in depletion of FRβ+ TAMs and controlled tumor progression in ovarian cancer, melanoma and colon adenocarcinoma (204). Ruella et al. found that CD123, the α chain of the receptor for IL-3, is expressed within Hodgkin lymphoma (HL) tumor masses both on cancer cells and on the M2-like TAMs. They demonstrated that CD123-specific CAR-T cells target both malignant cells and the surrounding immunosuppressive TME, and lead to the eradication of HL tumor xenografts. Moreover, anti-CD123 CAR-T immunotherapy induced long-term remission and the generation of an antitumor memory response. However, the use of immunodeficient mouse models in this studies does not enable for an accurate evaluation of the role of all endogenous immune system components (205).

New studies have underlined the importance of multiple antigen targeting as a means to both enhance the effectiveness of CAR-T cell therapy and to reduce off-target reactivity (206). To this end, Shu et al. generated CAR-T cells with two tandem CARs targeting CD47 and TAG-72 (Tumor-Associated Glycoprotein 72) (207). CD47 is a cell surface antigen highly expressed in ovarian tumors that functions equally as a macrophage “don’t eat me” signal enabling malignant cells to escape cell phagocytosis and thus detection by the immune system, by interacting with macrophage’ surface signal-regulatory protein-α (SIRPα) (208). TAG-72 is a pancarcinoma antigen and a tumor marker highly expressed in ovarian cancer (209). Blocking both CD47 and TAG-72 with CAR-T cells was associated with increased levels of macrophage-inflammatory protein (MIP)-1α and MIP-1β chemotactic factors in breast cancers, indicating functionality of the CD47 receptor in this model. The dual targeting strategy demonstrated enhanced ability of CAR-T cells to destroy tumor cells expressing low antigen levels, in favor of an increased binding avidity of the tandem CARs to the tumor cell. Another study, conducted by Xie et al., indicated that NanoCAR-T cells engineered to secrete anti-CD47 nanobodies (variable domain of heavy chain-only antibodies or V_H_H) were able to inhibit tumor growth, while avoiding toxicity encountered with systemic anti-CD47 therapy. This strategy of TAM reprogramming showed superior antitumor activity compared with standard CAR-T cells (210). The team also engineered anti-PD-L1 or anti-cytotoxic T-lymphocyte-associated protein 4 (CTLA-4) nanobodies secreting NanoCAR-T cells, which showed increased persistence. Moreover, this strategy to modify the intra-tumoral immune landscape by nanobody/V_H_H secretion can offer antitumor agents for multiple targets, has the advantage of being applied to immunocompetent animals and could limit systemic toxicity by means of local delivery at the tumor site (210).

Another approach to increase CAR-T cell infiltration and counteract the immunosuppressive TME is to induce tumor remodeling with adjuvant therapies (like chemotherapy or immune-checkpoint blockade). Srivastava et al. demonstrate that adding oxaliplatin to the lymphodepletion regimen given before ROR1 CAR-T cell infusion activates lung tumor macrophages to produce T cell-recruiting chemokines (reprogramming of TAMs to M1-macrophage). This results in improved CAR-T cell infiltration, tumor remodeling, and response to anti-PD-L1 checkpoint blockade, providing a strategy to improve CAR-T cell efficacy in the clinic. Moreover, a positive control loop has been noted in this model: CAR-T cells remodel the tumor microenvironment to amplify recruitment of endogenous T cells (211).

The aforementioned preclinical studies on CAR-T cells engineered to overcome the immunosuppressive effect of TAMs are summarized in the table below (**Table 5**).

**Table 5:** Summary of preclinical studies on CAR-T cells engineered to overcome the immunosuppressive effect of TAMs.

Targeted antigen	Type of cancer	Reference
mFRβ	Ovarian cancer	(203)
	Ovarian cancerMelanomaColon adenocarcinoma	(204)
CD123	Hodgkin lymphoma	(205)
CD47 & TAG-72	Ovarian cancer	(207)
CD47	MelanomaColon adenocarcinoma	(210)

#### 2.2.2 Tregs

**Figure 5:**
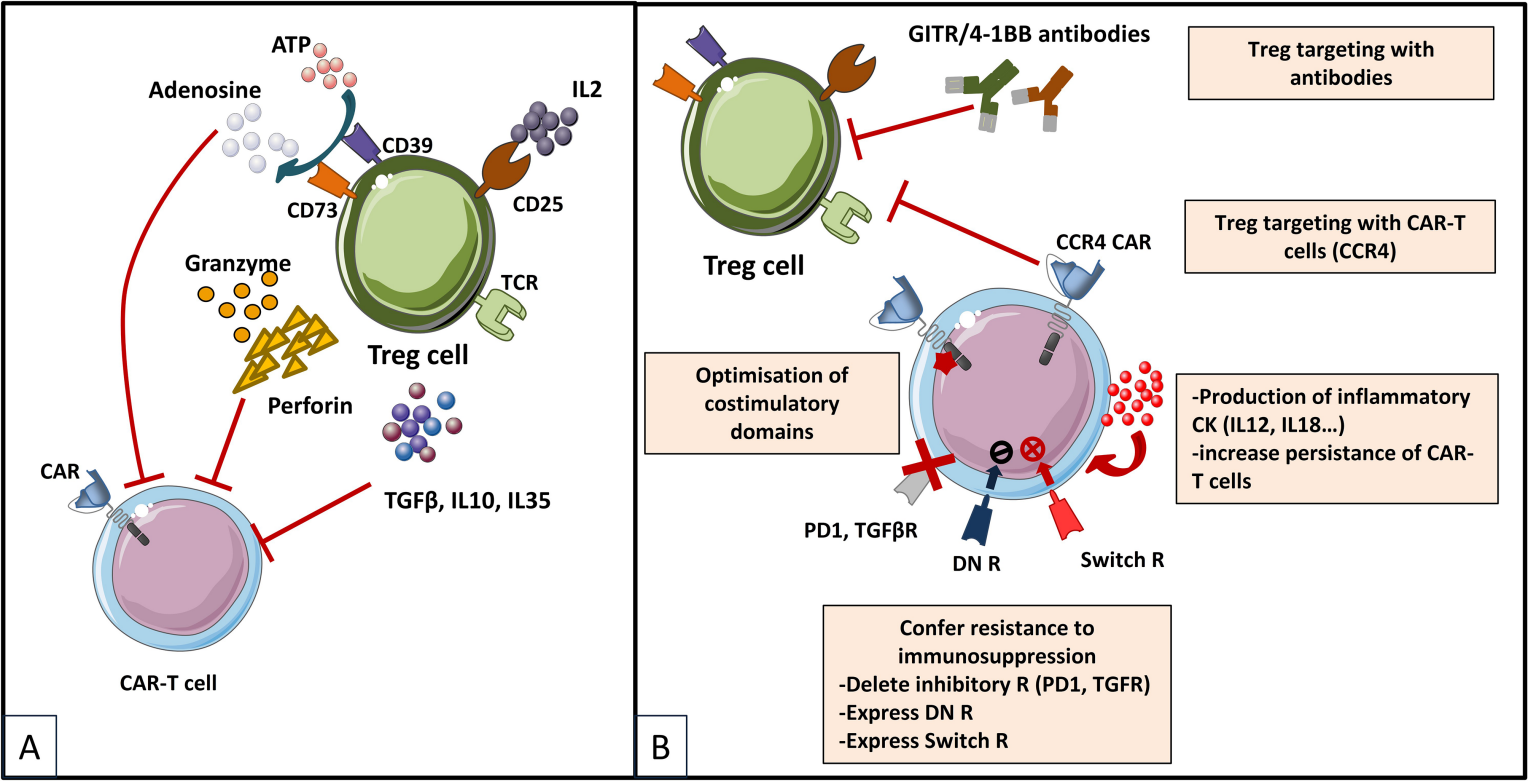
Immunosuppressive mechanisms exerted by Tregs in the TME **(A)** and engineering strategies to surmount Treg-induced immunosuppression **(B)** [Adapted from Togashi et al. (212), and Rodriguez-Garcia et al. (213)]. **(A)** depicts mechanisms for regulatory T (Treg) cells immunosuppressive effects on CAR-T cells based on their physiologic roles. Tregs are immunosuppressive cells highly dependent on IL-2. They bind to and deplete IL-2 from their surroundings, thus reducing availability to effector T (Teff) cells by constitutively expressing the high affinity IL-2 receptor (IL2R) subunit-α (CD25). Treg cells also produce immunosuppressive cytokines (IL-10, IL-35 and TGFβ), which can downregulate the activity of both Teffs and antigen presenting cells (APCs) and they exert direct cytotoxic effects by secreting granzymes and perforin. Moreover, Treg cells release large amounts of ATP, which is converted to adenosine (by CD39 and CD73) that can provide immunosuppressive signals to Teff cells and APCs. Other indirect mechanisms not depicted in the figure by which Tregs exert immunosuppressive effects are mediated by APC, as for instance Tregs expression of cytotoxic T lymphocyte antigen 4 (CTLA-4), which binds to CD80/CD86 on APCs, thereby transmitting suppressive signals to these cells and reducing their capacity to activate Teff cells. **(B)** shows therapeutic strategies to overcome the immunosuppressive TME sustained by Tregs. Some strategies are based on elimination of Tregs by CAR-T cells or combinations of CAR-T cells with monoclonal antibodies (mAbs) or drugs. CAR-T cells have been designed to target antigens expressed by Tregs for direct depletion. Other strategies are based on immunomodulation of the TME in order to increase CAR-T cells performance: 1) expression of proinflammatory cytokines by CAR-T cells and 2) optimization of costimulatory signaling domains in order to reduce IL-2 secretion and impair Treg expansion and tumor infiltration. Last type of strategies are meant to confer an intrinsic resistance to immunosuppression to CAR-T cells, either by endowing them with 1) dominant-negative receptors (DN R) meant to disrupt signaling, or 2) a chimeric switch receptor (CSR or Switch R) to convert negative signaling into a positive one, or by abrogating the expression of inhibitory receptors (like PD1 of TGFβ receptors) using genome-editing tools (knock out).

For years, regulatory T cells have been known to participate in the immunosuppressive environment of tumors. Due to their suppressive functions, Tregs are able to inhibit the effector functions of tumor-specific cells and reduce the effectiveness of active immunotherapy strategies based on the adoptive transfer of cytotoxic effectors. Therefore, several approaches have been developed to reduce the negative impact of Tregs in CAR-T cell therapies (**Figure 5**), and evaluated further on in various *in vitro* and pre-clinical studies (**Table 6**). Strategies to overcome immunosuppressive impact of the Treg population can be resumed as follows: (i) depletion strategies aiming to reduce cellular density of Tregs in the tumor, (ii) expression of interleukin receptors, hybrid interleukin receptors or switch receptors (iii) optimization of costimulatory domains of CAR-T cell, (iv) transgenic production of various cytokines by TRUCKs and, not least, (v) shielding of CARs from the suppressive effect of TGF-β by gene editing (**Figure 5**).

**Table 6:** Summary of preclinical studies on CAR-T cells to overcome the immunosuppressive effect of Tregs.

Targeted antigen	Expressed gene	Type of cancer	Reference
**A. Interleukin expression (TRUCK)**			
CD19	IL-12	Thymoma tumors	(214)
MUC16	IL-12	Ovarian cancer	(215)
GPC3	IL-12	HCC	(216)
CEA	IL-18	Pancreatic carcinoma	(217)
CD19	IL-18	Melanoma	(218)
MUC16	IL-18	Ovarian cancer	(219)
**B. Interleukin receptor expression**			
GD2	IL-7Rα	Neuroblastoma	(220, 221)
AXL	IL-7Rα	TNBC	(222)
**C. TGF-β targeting or inhibition**			
DNRII/sRII	–	Melanoma	(223)
PSMA	TGF-βRII	Prostate cancer	(224)
TGF-β	–	Melanoma	(225)
MSLN	*TGFBR2*-KO	MesotheliomaOvarian cancer	(226)
**D. TGF-β resistance**			
CCR8-DNR	CCR8	Pancreatic cancer	(64)
CEA	ΔCD28, IL-7 and IL-7Ra/IL-2Rb	Colon carcinoma	(227)
**E. Deletion of the LCK binding domain in CD28**			
FAP-F19	ΔCD28	Mesothelioma	(171, 174)
EGFRvIII	ΔCD28-4-1BB	Melanoma	(228)

CEA, Carcinoembryonic antigen.

For instance, the modification of CAR-T cells to produce IL-12 resulted in improved anti-tumor immune response by different mechanisms and in particular by decreasing CAR-T cells sensitivity to inhibition by regulatory T cells (214) but also by reduction of Tregs densities in the TME (215, 216). In a similar way, it was shown that CAR-T cells producing IL-18 promote antitumor immune responses (218, 219) by modifying the tumor environment notably by increasing the density of M1 macrophages and NK cells and by decreasing Treg infiltration, CD103+ suppressive DCs and M2 macrophages frequency (217, 219). It was also demonstrated that these two cytokines improve the antitumor response by increasing *in vivo* the survival and the proliferation of CAR-T cells that produce them (215, 216, 218). Promoting the proliferation of CAR-T cells *in vivo* is an important issue and initial strategies were based on injection of IL-2, a stimulator of T cells proliferation. Unfortunately, this adjuvant treatment has the major inconvenient of inducing the proliferation of Tregs in cancer patients. In order to overcome this side effect, some approaches have sought to increase CAR-T cells dependency on proliferative cytokines different from IL-2, such as IL-7. In various murine solid cancer models, the use of CAR-T cells expressing a constitutively active IL-7 receptor (IL7R) promotes *in vitro* activation, proliferation and cytotoxicity of CAR-T cells and increases survival of animals by eliciting a protective immune response (220–222). Moreover, unlike the case of IL-2 utilization, IL-7 conjoint injection upon CAR-T infusion does not result in increased proliferation and immunosuppressive function of Tregs, which have low expression of the IL-7R (220). On the other hand, endogenous production of IL-2 by activated T cells has a similar effect as exogenous IL-2 administration, and participates in the generation of Tregs in the TME. Therefore, in order to abrogate production of IL-2 by CAR-T cells, optimization of costimulatory domains of CARs, like the genetic modifications of the intra-cytoplasmic part of the CD28 molecule were designed (171, 174, 228). Preliminary results showed that the elimination of CD28-mediated IL-2 induction impairs CAR engraftment *in vivo*. However, when impairment of the IL-2 autocrine signaling is compensated for by another costimulatory molecule such as 4-1BB, the CARs accumulate in the bloodstream, suppress tumor growth and resist Tregs-induced immunosuppression (228). As IL-2 release and autocrine IL-2 receptor signaling seemed crucial in counteracting TGF-β repression, but CAR-T cell-released IL-2 negatively impacts the anti-tumor activity through sustaining survival and function of Treg cells, another elegant strategy to improve resistance to TGF-β is the engineering of a hybrid IL7/IL2 receptor to provide IL2 signaling upon IL7 binding. Therefore, Golumba-Nagy et al. designed TRUCKs releasing IL-7 and co-expressing hybrid IL-7Rα/IL-2Rβ receptor, which showed improved survival over a prolonged period and improved activity against TGF-β+ tumors (227).

Indeed, Tregs represent one of the major sources of TGF-β, an immunosuppressive cytokine which impacts the efficiency of immune effectors in the TME. Therefore, a different strategy to resist to TGF-β-induced immunosuppression is to inhibit or delete its receptor on the surface of CAR-T cells. Accordingly, the absence of a functional TGF-β receptor in CAR-T cells promotes proliferation as well as cytokine secretion, resistance to exhaustion and long-term *in vivo* persistence. Engineering methods for TGF-β receptor deletion/inhibition are: (i) the expression of a dominant negative (DN) receptor, and (ii) genetic disruption by gene-editing techniques like the CRISPR/Cas9 technology. To this regard, CAR-T cells expressing a dominant negative (DN) TGF-β receptor gene are more efficient at inducing protective responses (224, 225) and elimination of endogenous TGF-β receptor II (TGFBR2) in CAR-T cells using CRISPR/Cas9 technology reduces the induction of Treg cells and prevents CAR T cell depletion (226). These strategies aiming to use CAR-T cells modified to resist the immunosuppression induced by the TME derived TGF-β are currently undergoing clinical trials in hematological cancers and solid tumors such as prostate cancer (NCT03089203, NCT04227275.). The first clinical trial (NCT03089203) showed encouraging preliminary results (101) (**Table 10**).

In contrast to these strategies, which aim to reduce the negative impact of TGF-β on the anti-tumor response of CAR-T cells by inhibiting the expression of its receptor, other approaches are currently evaluating the therapeutic benefit of CAR-T cells modified to express a chimeric TGF-β receptor (switch receptor) whose activation by the cytokine would promote their functions (229). The aforementioned preclinical studies on CAR-T cells engineered to overcome the immunosuppressive effect of Tregs are summarized in the table below (**Table 6**).

#### 2.2.3 MDSC

MDSCs are a heterogeneous group of immature myeloid cells at various stages of differentiation and which differ from differentiated mature myeloid cells, such as neutrophils, macrophages, and dendritic cells (Dcs). As their name implies, MDSCs are a major group of immunosuppressive cells abundant in different types of cancers (230–233). Recent reports have suggested that MDSCs exert their immunosuppressive activity both on the innate and the adaptive immune system, both by cell-to-cell contact and by the secretion of soluble factors. MDSCs can also facilitate cancer progression by regulating cell mobility or even angiogenesis (234). MDSCs induce immunosuppression of T cell immune responses by various mechanisms: (i) degradation of amino acids essential for activation and proliferation (such as arginine, cysteine, or tryptophan) by production of arginase 1 (Arg1) and indoleamine 2,3-dioxygenase 1 (IDO1) enzymes (235), (ii) Treg induction *via* IL-10 and TGF- β secretion (236), (iii) suppression of T cell proliferation by MDSC-derived nitric oxide (NO) inhibition of the Jak/STAT5 pathway (237), (iv) impairment of T cell migration into tumor sites by the cleavage of the ectodomain of L-selectin by a disintegrin and metalloproteinase 17 (ADAM17) expressed by MDSCs, and reduction of E-selectin expression on endothelial cells caused by MDSC-derived NO (238), and (v) release of MDSC-derived reactive oxygen species (ROS) implicated in MDSC-mediated T cell suppression (239). In addition, accumulating research showed that MDSCs have potent mechanisms to promote cancer growth (via downregulation of IFN-γ and expression of MMP9) and metastasis (via TNFα, TGFβ, CXCL2 and S100A8/9) by establishing an immunotolerant environment (240). A meta-analysis, published by Zhang et al., concluded that the presence of MDSCs was correlated with poor prognosis in patients with solid cancer (241). Recent reports have also indicated that MDSCs may play a role in resistance to immunotherapy and CAR-T cell therapy (242).

**Figure 6:**
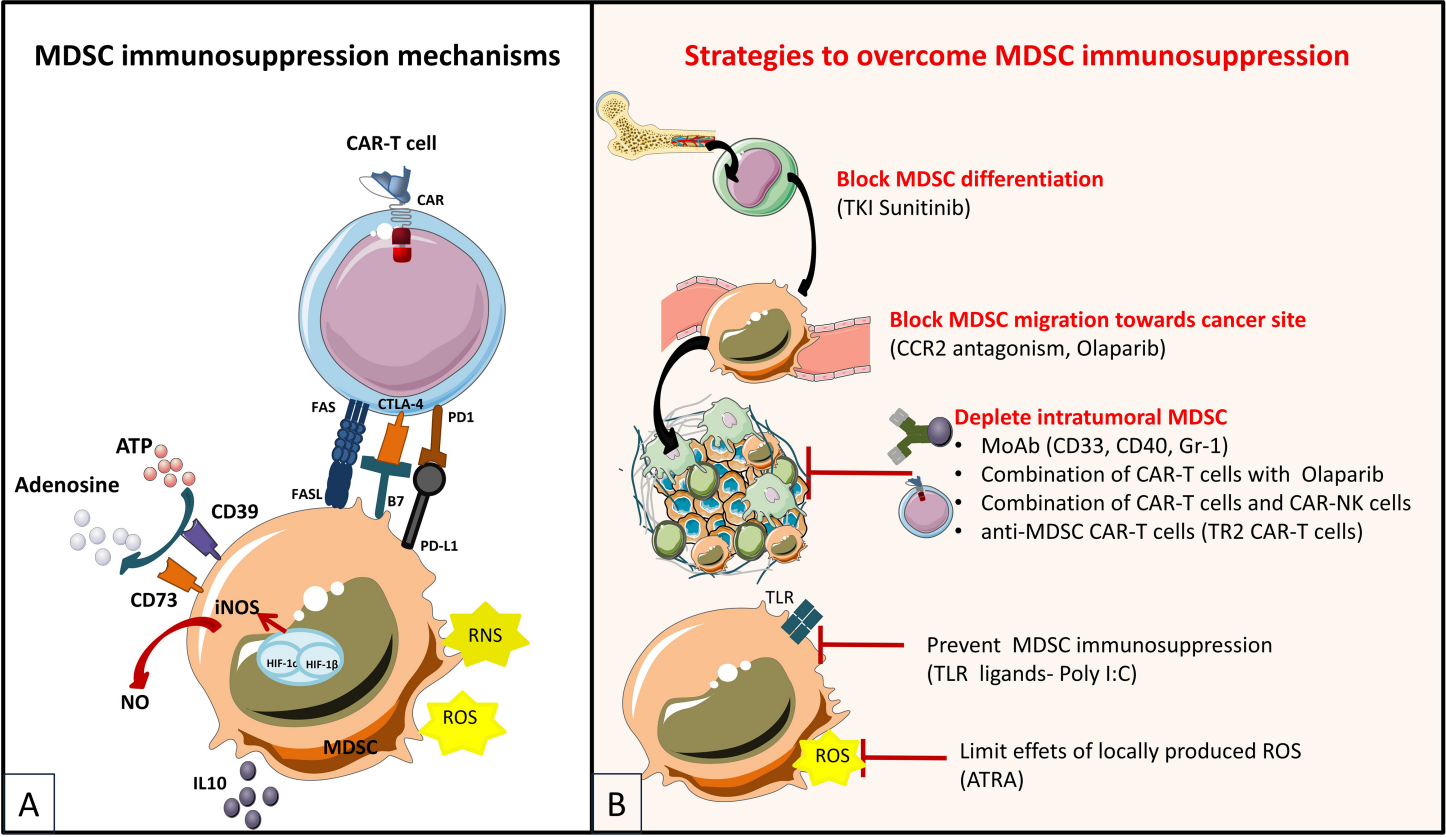
Immunosuppressive mechanisms exerted by MDSCs in the tumor microenvironment **(A)** and engineering strategies to surmount MDSC-induced immunosuppression **(B)** [Adapted from Krishnamoorthy et al. (250)]. **(A)** MDSC exert immunosuppressive effects in the TME (tumor microenvironment) by secretion of IL10 (which activates other immunosuppressive cells such as Tregs). Moreover, MSDCs can induce upregulation of checkpoint molecules (CTLA4, PD1) on T-cells, further inducing T-cell anergy, or can upregulate Fas which induces T-cell apoptosis by contact Fas/Fas-L mechanism. As an effect of hypoxia in the TME, MDSC can contribute to adenosine production by upregulation of CD73 and CD39. MDSCs also produce reactive oxygen (ROS) and nitrogen (RNS) species that can decrease T-cell proliferation and alter antigen recognition capabilities. **(B)** Strategies for targeting MDSCs in cancer are for example the prevention of MDSC differentiation from hematopoietic stem cells by the usage of Sunitinib, a tyrosine kinase inhibitor (TKI) that inhibits crucial factors for MDSC differentiation (VEGF and STAT3 activity). Second type of strategy is to prevent MDCS migrating to the tumor by targeting chemokine/chemokine receptor axes (CCR2/CCL2). Third, MDSCs depletion from the tumor can be achieved by using immunotherapy (depleting antibodies targeting CD33/gemtuzumab ozogamicin (GO), CD40 or Gr1) or chemotherapy. And least, mitigating the immunosuppressive effects of MDSCs at the tumor site can be realized by reducing the local effects of ROS (with ATRA or all-trans-retinoic acid) or by using TLR stimulation with specific ligands (TLR3 ligand polyinosinic-polycytidylic acid Poly I:C)

Several strategies have been deployed to increase CAR-T cells resistance to the immunosuppressive effects of MDSCs (**Table 7**). Among these various strategies, some are based on combinatorial therapies. Briefly, strategies aiming at counteracting MDSC are based on: (1) preventing differentiation and recruitment of MDSCs to the tumor bed, (2) depleting tumor infiltrating MDSCs or (3) mitigating MDSCs immunosuppressive effects (**Figure 6**). For instance, blocking of MDSC differentiation could be obtained by using a multitargeted TK inhibitor (TKI), Sunitinib, which inhibits STAT3 signaling and induces apoptosis of murine MDSCs. Li et al. demonstrated that coadministration of carbonic anhydrase IX (CAIX)-CAR-T cells with Sunitinib significantly enhanced therapeutic efficacy against a mouse model of human metastatic renal cancer and resulted in prolonged survival of mice, as well as reduction in the number of MDSCs at the cancer site (246). Sunitinib has already shown positive results in a clinical trial (NCT03277924), in combination with anti-PD1 blocking in advanced sarcoma (251) (**Table 10**).

**Table 7:** Summary of preclinical studies on CAR-T cells strategies counteracting the immunosuppressive effect of MDSCs.

Targeted antigen	Expressed genes	Co-administered agent	Type of cancer	Reference
GD2	–	ATRA	Osteosarcoma	(243)
GD2MSLNEGFRvIII	–	GO	Solid tumors	(244)
EGFRvIII	–	Olaparib	Breast cancer	(245)
CAIX	–	Sunitinib	Metastatic renal caner	(246)
EGFRvIII	–	Poly I:C	Breast cancerColon cancer	(247)
GD2	–	NKG2D.ζ CAR-NK	Neuroblastoma	(248)
MUC1	TR2.4-1BB	–	Breast cancer	(249)
HER2	TR2.4-1BB	–	Breast cancer	

Blocking recruitment of MDSC to the tumor bed has been obtained for example by impeding the chemotaxis axis SDF1α/CXCR4 by Sun et al. They proved that olaparib, a Poly(ADP-ribose) polymerase inhibitor (PARPi), could enhance the anti-tumor immune response of EGFRvIII-specific CAR-T cells as well as the recruitment of CD8+ T cells in mouse breast cancer models. Moreover, mice treated with a combination of olaparib and EGFRvIII-specific CAR-T cells showed decreased expression of SDF1α (CXCL12), one of the main MDSC chemoattractant, as well as decreased MDSC recruitment. In parallel, a decrease of the CXCL12 receptor, CXCR4, expression has been noted in MDSCs treated with olaparib, proving that Olaparib might reduce MDSC recruitment by interfering with the SDF1α/CXCR4 axis (245). Other strategies to mitigate MDSCs recruitment to the tumor bed could by the blocking of MDSCs chemokine receptors, as it is the case of CCR2. The CCR2 blocking technique has already been employed but, it can, unfortunately also impede TILs recruitment to the tumor bed (252, 253). Even so, combination of CCR2 blockade with anti-PD1 therapy enhanced anti-tumor responses in many preclinical cancer models (melanoma, breast cancer) (254). Moreover, CCR2 antagonism decreased MDSC counts in pancreatic cancer patients (NCT02345408) (255) (**Table 10**).

Other strategies already deployed to deplete intra-tumoral MDSC were antagonism or inhibition of specific receptors, like Gr1, CD40 (with mAbs) or CD33 (256, 257). CD33 was identified as a common surface marker of MDSCs and Fultang et al. provided evidence that Gemtuzumab ozogamicin (GO), an antibody-drug conjugate consisting of a humanized mAb targeting CD33 linked to an intracellular toxin named calichamicin, can eliminate MDSCs, leading to CAR-T cell reactivation against multiple cancers. In this study, coadministration of GO restored GD2, MSLN and EGFRvIII CAR-T cell proliferation, leading to increased tumor cell death (244). Moreover, depletion or expansion reduction of MDSC in combination with CAR-T cell therapy has also been employed by using GM-CSF or PD-L1 neutralization as it has been recently shown the immunosuppressive capacities of MDSCs are modulated by GM-CSF through the PD1-PD-L1 axis (258). In a different approach, Parihar et al. demonstrated the effectiveness of specific CAR-NK cells in suppressing MDSCs (248). It has been reported that MDSCs overexpress NKG2D ligands, which are able to activate the NKG2D cytotoxicity receptor on NK cells (259). The immunosuppressive TME restricts, however, NK cell activation. To overcome this obstacle and enhance MDSC depletion *in vivo*, they generated a CAR that fuses the NKG2D receptor to CD3ζ, the NKG2D.ζ CAR-NK cells. Coadministration of these NKG2D.ζ CAR-NK cells increased the anti-tumor activity of GD2 CAR-T cells in a xenograft model of neuroblastoma (248).

In a 2021 ASCO annual meeting’s abstract, Nalawade et al. demonstrated that MUC1-specific CAR-T cells engineered with a novel chimeric co-stimulatory receptor, TR2.4-1BB, comprising a ScFv derived from a TNF-related apoptosis-inducing ligand receptor 2 (TR2) mAb with a 4-1BB endodomain., induced MDSC apoptosis. As MDSCs express TR2, this strategy could warrant selective MDSC depletion. MUC1.TR2.4-1BB CAR-T cells showed increased cytotoxic activity against breast cancer tumors and inhibited tumor growth more effectively than either MUC1 CAR-T cells or TR2.4-1BB T cells. Similar results have been observed with HER2.TR2.4-1BB CAR-T cells in a HER2+ breast cancer model (249).

Furthermore, Di et al. proved that administration of the toll-like receptor 3 (TLR3) ligand polyinosinic-polycytidylic acid (poly I:C) can increase EGFRvIII-specific CAR-T cell efficacy in immune competent mice bearing colon and breast cancers by enhancing specific lysis of cancer cells and cytokine release upon antigen stimulation. Poly I:C also impeded the suppressive effect of MDSCs on T cell proliferation (247)

And finally, Long et al. identified all-trans-retinoic acid (ATRA) as an effective agent in decreasing the suppressive effect of MDSCs. Co-treatment with ATRA and GD2 CAR-T cells led to an increased antitumor activity compared to ATRA or CAR-T cells treatment alone. These positive effects of ATRA treatment can be explained by an augmented expression of glutathione synthase in MDSCs resulting in higher glutathione synthesis and neutralization of ROS (which contribute to T cell depletion and impede MDSC differentiation) (243).

#### 2.2.4 Enhancing Persistence/Fitness of Genetically Modified T Cells by Interleukin Production

In order to increase persistence and/or maintenance in the immunosuppressive TME, CAR-T cells can be genetically engineered to produce vital cytokines. This engineering strategy is meant to complete the third signal of the immunological synapse (i.e. cytokine stimulation), which is lacking or insufficient in the TME. Various cytokines have already been transgenically expressed in CAR-T cells, the most common being: IL-7, IL-12, IL-15, IL-18, IL-21 and IL-23 (**Table 8**). Some of them hace been addressed in the Treg section (see section 2.2.2 on Tregs). For an extensive review on gene-edited interleukin CAR-T cells published recently, see Zhang et al. (268).

**Table 8:** Summary of preclinical studies on CAR-T cells engineered to produce vital cytokines or to express transgenic cytokine receptors.

Targeted antigen	Expressed cytokines/Cytokine receptors	Type of cancer	Reference
MUC16	IL-12	Ovarian cancer	(260)
CEA	IL-18	Advanced pancreatic cancerAdvanced lung cancer	(217)
CLL1	IL-15	AML	(261)
VEGFR2	IL-15	Melanoma	(262)
IL-13Rα2	IL-15	Glioma	(263)
GD2	IL-15	Neuroblastoma	(264)
GPC3+	IL-15 & IL-21	HCC	(265)
GD2	p40 of IL-23R	Neuroblastoma	(266)
PSMA & IL-23	–	Prostate cancer	(267)
GD2	IL-7R	Neuroblastoma, Glioblastoma	(221)

Therefore, another strategy to overcome the immunosuppressive TME, is the use of fourth generation CAR-T cells genetically redirected for antigen-unrestricted cytokine-initiated killing (TRUCKs), modified to secrete immune stimulatory cytokines (2). Yeku et al. reported the efficacy of IL-12 secreting TRUCKs directed against mucin-16 (MUC16), known as 4H1128ζ-IL-12 T cells, in an aggressive disseminated mouse ovarian cancer model. Indeed, these CAR-T cells induced the eradication of TAMs *via* Fas/FasL pathway, secreted more inflammatory cytokines (such as IFN-γ) and exhibited increased cytotoxicity *in vitro* and *in vivo*. Moreover, cytokine stimulation of 4H1128ζ-IL-12 T cells was associated with increased resistance of CAR-Ts in the TME. The armored CAR-T cells also showed a decreased expression of eomesodermin (Eomes), forkhead box P3 (FOXP3), CTLA-4, LAG-3, TIM-3 and programmed death-ligand 1 (PD-L1), all of which play a major role in the establishment of an immunosuppressive environment (260). Similarly, Chmielewski et al. found that treatment with IL-18 secreting TRUCKs directed against carcinoembryonic antigen (CEA), enhanced CAR-T cell function and survival of mice with advanced pancreatic and lung cancers, and induced an acute Th1 inflammatory response. Data showed an increase in the number of NK cells and M1-like macrophages, and a decrease in the number of M2-like macrophages, Tregs, and inhibitory DCs, allowing for an enhanced antitumor activity (217). The interleukin IL-15 emerged as an immunomodulatory cytokine with anti-tumor effects thanks to its roles in inducing expansion and activation of NK, natural killer T (NKT) cells, and long-lasting memory CD8+ T cells (CTLs). Indeed, IL-15 promotes memory CTL survival and effector function (cytotoxic activity and IFN γ release) and could prevent Tregs from influencing the effector functions of CD4 and CD8 T cells. Moreover, IL-15 can inhibit IL-2 activation induced cell death of effector lymphocytes (269). To this regard, many groups found that preconditioning with IL-7 and IL-15 resulted in better *in vitro* expansion of CAR-T cells as well as superior antitumor effects *in vivo* and even increased efficacy upon immune-checkpoint blockade (increased CAR T cell responses to anti-PD-1 adjuvant therapy) (262, 270–272).

Therefore, transgenic expression of IL-15 seemed like an appealing strategy to enhance CAR-T cell effector function, by enhancing proliferation and persistence of CAR-T cells in the TME and has been used in preclinical models of acute myeloid leukemia (AML), melanoma, glioblastoma or neuroblastoma (261–265, 273). In preclinical models of AML, CLL1-directed CAR-T cells with transgenic expression of IL-15 showed increased expansion, survival and antileukemic potency. Unfortunately, co-expression of IL-15 was associated with lethal cytokine release syndrome (CRS), a fatal adverse effect that could be prevented with anti-TNF-antibodies pretreatment and depletion of IL-15 secreting CARs by the inducible caspase-9 (iCas-9) suicide switch (261).

Another fourth generation CAR targeting VEGFR2 on the tumor vasculature and co-expressing murine IL-15 has been evaluated as a tool to overcome TME immunosuppression in immunocompetent, syngeneic melanoma-bearing mice. As in previous studies, expanded CAR-T cells transduced to co-express IL-15 cultivated with both IL-7 and IL-15 showed enhanced expansion as well as a TCM cell phenotype predominantly. *In vivo*, CAR-T cells up-regulated the antiapoptotic marker Bcl-2 and down-regulated the inhibitory receptor PD-1. These CARs showed enhanced effector functions, engraftment and tumor control, in part through reprograming of the TME in favor of protective endogenous immunity, including NK cell activation and reduced presence of M2 macrophages (262). IL-15 co-expression was also used in a model of IL-13Rα2-positive glioma (IL-13Rα2-CAR.IL-15 T cells) and results showed better *in vivo* persistence and greater antiglioma activity. Unfortunately, despite enhanced recognition of glioma cells, greater proliferative capacity and increased production of cytokines, the improved T cell persistence was associated with recurrence of gliomas with down-regulated IL-13Rα2 expression. Therefore, at least in GBM treatment, this engineering strategy should be coupled with multiple antigen targeting techniques (263). Expression of IL-15 as a reinforcement proliferative signal was also used in a neuroblastoma model. As in previous studies, GD2 specific CARs armored with IL-15 (GD2.CAR.15-Ts) showed reduced expression of the PD-1 receptor as well as superior antitumor activity. IL-15 forced expression resulted in enrichment in stem cell-like cells (TSCM-like). CAR-Ts were engineered to contain the inducible caspase 9 (iCas9) safety switch (264).

Another method of interleukin gene-editing of CAR-T cells is the co-expression of two simultaneous cytokines. Batra et al. engineered fourth generation CAR-T directed against GPC3+ composed of a 4-1BB costimulatory motif and co-expressing both IL-21 and IL-15 and found superior expansion and antitumor activity against HCC in a preclinical model. IL-21 and IL-15 armored GPC3+ CAR-T cells showed higher proliferation at least in part by maintaining the expression of T cell factor-1 (TCF-1), a transcription factor critical for T cell development and survival. Moreover, manufacturing outcome showed a higher percentage of TSCM and TCM populations. For effective management of toxicity risk in the clinical setting, the authors also proved that the iCas-9 “suicide switch” can effectively eliminate these CAR-T cells. Two clinical trials were open to explore anti-tumoral benefit: NCT02932956 and NCT02905188 (265) (**Table 10**).

Nonetheless, co-expression of cytokines by genetically engineered CAR-T cells as a mean of boosting anti-tumor activity has the inconvenience of constitutive cytokine signaling in T cells and activation of bystander cells which may cause toxicity. To prevent hyper-activation and excessive cytokine production of CAR-T cells, another team engineered CAR-Ts containing signaling TCR-responsive nanoparticles containing human IL-15 superagonist complex. These protein nanogels embedded in CARs were associated with enhanced selective expansion of CAR-T cells within tumors and improved therapeutic efficacy (273).

In order to circumvent interleukin forced expression, Xincong et al. engineered CAR-T cells expressing the p40 subunit of the IL-23 receptor (p40-Td cells). The p40 subunit of the IL-23R is the only subunit which is not up-regulated upon TCR stimulation. Over-expression of the p40 subunit induced selective proliferation of CAR-T cells *via* an IL-23 autocrine loop. Moreover, p40-Td cells showed improved antitumor capacity both *in vitro* and *in vivo* as well as attenuated side effects in comparison to CAR T cells expressing IL-18 or IL-15 (266).

The interleukin IL-23 has also been targeted with a different strategy, in the context of prostate cancer, by designing CAR-Ts targeting both PSMA and IL-23 (IL-23 mAb). The inflammatory cytokine IL-23 plays an active role in tumorigenesis, by upregulating certain MMPs (MMP9), by increasing angiogenesis and infiltration of M2 macrophages and neutrophils and by reducing CD8 T cell infiltration in the TME. Moreover, it has been proven that IL-23 secreted by MDSCs drives castration-resistant prostate cancer by activating the androgen receptor pathway (274). Therefore, Wang et al. designed three types of CAR-T cells in order to simultaneously target PSMA expressing cells and capture local soluble IL-23 produced by tumor cells or by MDSCs: either (i) dual or duo CAR-Ts expressing 2 CARs at the surface (IL-23mAb-T2A-PSMA), or (ii) a tandem CAR IL-23mAb/PSMA or (iii) PSMA-CARs secreting anti-Il-23 Ab. IL-23 and PSMA targeted duo-CAR-Ts (IL-23mAb-T2A-PSMA) were more efficient in prostate cancer eradication than PSMA CARs only and induced stronger T cell activation, and increased cytokine production when compared to single-molecule tandem CAR IL-23mAb/PSMA (267).

In a different approach, Shum et al. engineered CAR-T cells constitutively expressing the IL-7 receptor (C7R CAR-T cells), in order to deliver potent stimulation and increase CAR-T persistence and antitumor activity (221). An ongoing clinical trial evaluates efficacy of C7R-GD2.CAR T cells in the treatment of brain tumors (NCT04099797) (**Table 10**). Except armored CAR-T cells secreting IL7 and/or IL2 cited above (see *Overcoming the Mismatch or the Dysregulation of Chemokine Receptor/Ligand Axes*), armored CAR-T cells secreting other survival cytokines have also gone to the clinic (like IL-15 and/or IL-21: NCT04715191, NCT05155189, NCT02932956 and NCT02905188, NCT03721068, NCT04377932, and NCT05103631) (**Table 10**).

#### 2.2.5 Overcoming Inhibition by Negative Immune Checkpoints

The tumor-related immune response is regulated by various stimulating and inhibitory signals. Immune checkpoints (ICs) insure the maintenance of immune homeostasis, and thus self-tolerance, by regulating the time course and the intensity of the immune reaction. However, receptor-based signal cascades emerging from ICs play a negative regulatory role in T cells, by inducing immune tolerance and therefore tumor escape from immunosurveillance (275). The first main ICs identified as essential receptors for T cell and CAR-T cell inhibition and apoptosis are CTLA-4 and PD-1 (276). Other immunoreceptors extensively studied in cancer are LAG-3, TIGIT, T-cell immunoglobulin and mucin containing protein-3 (TIM3) and B and T lymphocyte attenuator (BTLA). Since, many different monoclonal Abs (mAbs) and bispecific antibodies (BsAbs) that prevent ligand- inhibitory IC receptor engagement have been used to block immune checkpoints. IC receptors use mono-tyrosine signaling motifs, such as ITIM and immunoreceptor tyrosine-based switch motifs (ITSM), to exert their inhibitory activity (276). IC inhibition either in monotherapy or as supplementary therapy turned out to be a very efficient weapon to fight cancer (277). As PD1/PD-L1 inhibition is the most studied axis, this chapter mainly focuses on PD-1/PD-L1 inhibition in CAR-T cell therapy (**Table 9**).

**Table 9:** Summary of preclinical studies on CAR-T cells to overcome the inhibitory effect of ICs.

Targeted antigen	Expressed genes	Co-administered agent	Type of cancer	Reference
HER-2	–	Anti-PD-1	Breast cancer	(278)
GD2	–	Pembrolizumab	NeuroblastomaMelanoma	(279)
FAP	–	Anti-PD-1	Malignant pleural mesothelioma	(174)
MUC16	PD-1 scFv	–	Ovarian cancer	(280)
EGFR	PD-1 scFv	–	Gastric cancer	(281)
CAIX	PD-L1 scFv	–	RCC	(282)
MSLN	PD-1 DNR	–	Malignant pleural mesothelioma	(283, 284)
MSLN	PD1/CD28switch-receptor	–	Mesothelioma	(285)
PSCA		–	Prostate cancer	
CD133	PD-1 KO	–	Glioma	(286)
MSLN	PD-1 KO	–	TNBC	(287)
GPC3	PD-1 KO	–	HCC	(288)
EGFRvIII	PD-1 KO	–	GBM	(289)

PD-1 is a member of the B7/CD28 family which exerts its role in modulating T cell activity by interacting with two ligands- PD-L1 and PD-L2. PD-1/PD-L1 binding impedes the synthesis of IFN-γ and IL-2, which decreases T cell proliferation (290). Furthermore, the overexpression of PD-L1 is correlated with poor prognosis in many cancers (291–294). In order to block the interaction between PD-1 and PD-L1, various mAbs, such as nivolumab, pembrolizumab, avelumab, lambrolizumab, and atezolizumab, have been developed (295). After encouraging results from different clinical trials, the FDA granted accelerated approval to nivolumab for the treatment of advanced melanoma in 2014 (296), and advanced squamous NSCLC in 2015 (297). Nivolumab has also shown positive results in many other cancers, such as R/R HL (298) and HCC (299). Furthermore, other anti-PD-1 mAb, pembrolizumab (humanized IgG4 kappa anti-PD-1 mAb), has been approved by the FDA for the treatment of many types of cancer, including unresectable or metastatic melanoma in 2014 (300), advanced NSCLC in 2015 (301), recurrent or metastatic HNSCC in 2016 (302), and locally advanced or metastatic urothelial carcinoma in 2017 (303).

**Figure 7:**
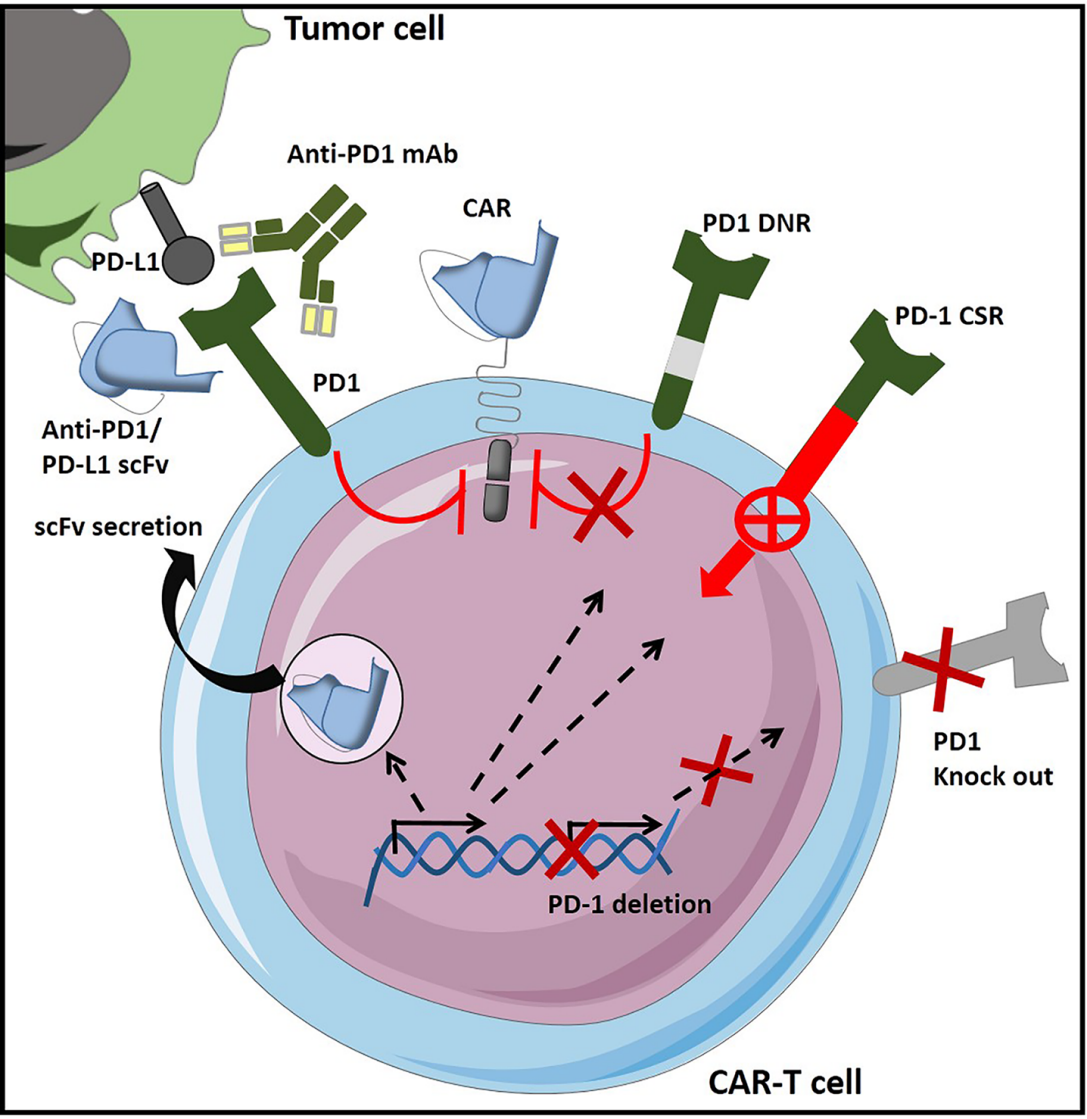
CAR-T cell engineering strategies to overcome inhibition form negative immune checkpoint regulation – Example of PD1/PD-L1 axis targeting in CAR-T cells [Adapted from Rafiq et al. (31)]. In order to prevent CAR-T cell exhaustion and immunosuppression in the TME, different strategies can be used, like combination of CAR-T cells with immune checkpoint inhibitors (ICIs like anti-PD1 or PD-L1 antibodies). PD1-medited inhibition can also be surmounted by designing CAR-T cells that secrete either PD-1-blocking or PD-L1 blocking scFv. Other means of shielding CAR-T cells from the inhibitory effect of the PD1/PD-L1 interaction is to design genetically modified CAR-T cells that express a dominant negative PD-1 receptor (PD-1 DNR) which interferes with PD1 downstream signaling or a PD-1 chimeric switch receptor (CSR), which converts an inhibitory signaling into an activating one. Last type of strategy is based on PD1 expression deletion either by genetic knock-out or by means of shRNA (short hairpin RNA) inhibition.

Given acknowledged CAR-T cell dysfunction following engagement of IC receptors and especially spectacular results obtained with anti-PD1 and anti-PD-L1 ICI mAbs, different strategies were deployed in order to enhance CAR-T cell efficacy (**Figure 7**).

John et al. were the first to demonstrate that inhibiting an important immunosuppressive pathway such as PD-1 can significantly increase adoptive immunotherapy efficacy using genetically modified T cells (278). To this regard, PD-1 expression was shown to be significantly enhanced on CAR-T cells cocultured with PD-L1+ HER-2+ tumor cells, whereas PD-1 inhibition enhanced CAR-T cell proliferation and activity *in vitro* and *in vivo*. Moreover, the coadministration of anti-PD-1 mAbs together with HER-2 specific CAR-T cells enhanced HER-2+ tumor regression and mice survival in a transgenic animal model without any sign of autoimmunity. Interestingly, CAR-T cell and anti-PD-1 mAbs combined therapy significantly depleted MDSCs but not Tregs at the cancer site, as compared to untreated control mice. Similar results have been observed by Gargett et al. with GD2-specific CAR-T cells combined to pembrolizumab against neuroblastoma and melanoma cell lines (279), and Gulati et al. with FAP-specific CAR-T cells combined to anti-PD-1 mAbs against a malignant pleural mesothelioma cell line (174). Many ongoing clinical trials are evaluating the efficacy of the administration of CAR-T cells with anti-PD-1/PD-L1 blocking antibodies in patients with solid tumors (NCT04995003, NCT02414269, NCT01822652, NCT04003649, NCT03980288, and NCT03726515).

In order to avoid repeated anti-PD-1/PD-L1 mAbs administration and the toxicity associated with it, new approaches have been proposed to inhibit the PD-1/PD-L1 axis, by engineering CAR-T cells expressing PD-1-blocking scFv (280, 281) or PD-L1-blocking scFv (282), expressing PD-1 DNR (283, 284), or chimeric switch receptors (CSR, like PD1/CD28 CSR) (285) (**Figure 7**). Various ongoing clinical trials are evaluating anti-PD-1 antibodies secreting (NCT03030001, NCT03615313, NCT04489862, and NCT02873390), anti-PD-L1 scFv secreting (NCT04556669) or PD-1 nanobodies secreting CAR-T cells (NCT04489862, NCT04503980 and NCT05089266) (**Table 10**). Genetic engineering strategies aiming at counteracting immunosuppressive signaling by the design of PD1/CD28 CSR are also evaluated in the clinical setting (clinical trials NCT03932955, NCT04850560, NCT02937844). Other clinical trials evaluate efficiency of CAR-T cells designed to resist immunosuppression by various other mechanisms, employing mutant PD-1 proteins, PD-1 dominant negative receptors (DNR), cytoplasmic activated PD-1 or PD-1 downregulation (NCT03540303, NCT04768608, NCT04577326, NCT04163302, NCT04162119, and NCT04836507) (**Table 10**).

**Table 10:** Summary of clinical trials on CAR-T cells designed to improve tumor homing and penetration, CAR-T cell persistence and resistance to immunossupresssion in solid tumors.

Type of strategy	Targeted antigen	Additional engineering strategies	Type of cancer	Othertherapy	Phase/NCTnumber	Number of patients	Preliminary outcomes	Reference
Homing	–	CXCR2 and NGFR Expression	Metastatic melanoma	Lymphodepletion (cyclophosphamide & fludarabine),High-dose Aldesleukin (rhIL2)	Phase I/II NCT01740557	Active, not recruiting3/36 enrolled	None posted	(304)
Homing, Maintenance & Stroma targeting	Nectin4 FAP	IL-7 and CCL19/IL-2 production	Nectin4+ advanced NSCLC, breast, bladder, OC or PC	Intravenous minimally invasive surgery	Phase I NCT03932565	Recruiting	None posted	–
Homing, Maintenance & Protection from IS	MSLN GPC3, and/or TGFβ	CCL19 expression, IL7 production and/or scFv against PD1/CTLA4/TIGIT +/-PD1 KO	Advanced HCC/ PC/OC	Lymphodepleting chemotherapy	Phase I NCT03198546	6 patients	2 CR, 2 PR and 2SD No high grade toxicities	(68)
Stroma targeting	FAP	–	Malignant pleural mesothelioma	Anti-PD1 (in 1/3)	Phase I NCT01722149	3 patients	2/3 alive at 18 months follow-up	(169)
Neovasculature targeting	VEGFR2	–	Metastatic melanoma or renal cancer	Non-myeloablative lymphodepletion (fludarabine & cyclophosphamide) + Aldesleukin (rhIL-2)	Phase I/II NCT01218867	24 patients	Lack of objective responses: 1/24 PR, 1/24 SD, 22/24 PD Grade 3/4 toxicity in 5/24	–
	PSMA	–	Prostate cancer	Non-myeloablativeconditioning,Low/moderate dose of IL-2	Phase I NCT00664196	Suspended	None posted	–
	PSMA	HSVtk gene for ganciclovir elimination	Metastatic CRPC	Cyclophosphamide	Phase I NCT01140373	Active, not recruiting7 patients	2/7 SD, 2/7 PD	(305)
	PSMA	–	Metastatic prostate cancer	Non-Myeloablative Conditioning (fludarabine & cyclophosphamide) Low dose of IL-2	Phase I/II NCT01929239	Suspended	2/5 PR	(306)
Antigen Escape Prevention or increased immune engagement/ Bispecific or Dual CARs	c-Met/PD-L1	–	Primary HCC	–	Early Phase I NCT03672305	Recruiting	None posted	–
	B7H3CD19	Suicide mechanisms: EGFRt or Her2tG	Pediatric Solid Tumor	+/- Cetuximab (anti- EGFRt) or Trastuzumab (anti-HER2tG)	Phase INCT04483778	Recruiting	None posted	–
	EGFRCD19	Suicide mechanisms: EGFRt or H2tGand express a EGFR-specific and a CD19-specific receptor	R/R Solid Tumors in Children and Young Adults	–	Phase I NCT03618381	Recruiting	None posted	–
	HER2PD-L1	–	Pleural or Peritoneal Metastasis of HER2+ Cancer	–	Early Phase INCT04684459	Active, not recruiting	None posted	–
Potentiating activation/ Switchable CARs	PSCA	Inducible MyD88/CD40 co-activation switch	Metastatic prostate and pancreatic cancer	Fludarabine & cyclophosphamide Rimiducid (inducible MyD88/CD40 co-activation switch)	Phase I/IINCT02744287	RecruitingPreliminary results in 15 patients	8/15 SD, 3/15 PD, No CRS, 1/15 serious Grade 2 AE	(307)
Universal CARs	–	UniCAR02-T Cells and recombinant antibody derivative TMpPSMA	Prostate Cancer	Fludarabine & cyclophosphamide	Phase I NCT04633148	Recruiting	None posted	–
Protection from IS by PD-1 gene deletion/gene expression inhibition	MSLN	CRISPR-Cas9 MediatedPD-1 and TCR/CD3 Gene-knocked Out(PDCD1 and TRAC KO)	MSLN+ metastatic solid tumors	No prior lymphodepletion	Phase I NCT03545815	15 patients	2/15 SDNo dose-limiting toxicity or unexpected AEs	(308)
	MUC1	CRISPR-Cas9 PD-1 gene KO	Advanced NSCLC(IIIb to IV)	PD-1 mAb (PD-1 antibody treated group) or sham (control)	Phase I/IINCT03525782	Recruiting(Preliminary results on 20 patients)	11/20 SD, 9/20 PD,no grade 3-5 AEs, no CRS	(309)
	–	CRISPR-Cas9 PD-1 gene KO	Advanced prostate cancer	–	Phase I/II NCT03525652	Unknown status	None posted	–
	–	CRISPR-Cas9 PD-1 gene KO	Metastatic NSCLC	Cyclophosphamide	Phase I NCT02793856	22 enrolled/12 treated	2/12 SD, no PR, 11 cancer progression related deaths,Grade 1/2 AEs	(310)
	–	CRISPR Cas9 PD-1 gene KO	Advanced ESCC	Hydrocortisone	Phase INCT03081715	21 enrolled/17 treated	6/17 SD, 11/17 PD (10 cancer progressionrelated deaths), no PR,no grade 3/4 AEs	(311)
	Cancer-specific TCR transgene (NY-ESO-1)	NY-ESO-1 TCR expressions and multiplex CRISPR-Cas9 editing (KO of endogenous TCR and PD-1)	MM, sarcoma(SS, MRCL)	Lymphodepleting chemotherapy (Cyclophosphamide & Fludarabine)	Phase INCT03399448	3/4 infused	2/3 SDNYCE T cells detectable in circulation for up to 9 months (1 MM)	(312)
	–	PD-1 gene KO	Metastatic Renal Cell Carcinoma	CyclophosphamideIL2	Phase I NCT02867332	Withdrawn (No funding)	None posted	–
	EBV-specific autologous CTLs	CRISPR-Cas9 PD-1 gene KO	EBV+ Stage IV cancers: Gastric and Nasopharyngeal Carcinoma, TCL, Adult HL and DLBCL	Fludarabine Cyclophosphamide IL-2	Phase I/IINCT03044743	Recruiting	None posted	(313)
	–	CRISPR-Cas9 PD-1 gene KO	Invasive Bladder Cancer (Stage IV)	Cyclophosphamide IL-2	Phase I NCT02863913	Withdrawn (No funding)	None posted	–
	MSLN	CRISPR-Cas9 PD-1 gene KO	MSLN+ multiple solid tumors	Paclitaxel,Cyclophosphamide+/- Gemcitabine	Phase INCT03747965	9 treated (6 pancreatic, 2 ovarian and 1 colorectal cancer)	7 evaluable patients:2/7 PR, 4/7 SD,2/7 grade 1 CRS	(314)
	MUC-1	CRISPR-Cas9 PD-1 gene KO	Advanced esophageal cancer/ESCCNSCLC (IIIb to IV)	–	Phase I/IINCT03706326	8 enrolled	5/8 SD, 3/8 PD,no grade 3-5 AEs, no CRS	(315)
		PD-1 gene KO	Refractory Thyroid Cancer	–	Phase I ChiCTR1900022620**	Not yet recruiting	None posted	–
		PD-1 gene KO	Advanced, recurrent or metastatic solid tumors	–	Phase I ChiCTR-OIN-17012136**	Recruiting	None posted	–
		PD-1 gene KO	Lung cancer	–	ChiCTR1800016023**	Not yet recruiting	None posted	–
	MUC-1	PD-1 gene KO	MUC1+advanced breast cancer	–	Phase 0 ChiCTR1900025088**	Recruiting	None posted	–
Protection from IS by immune-checkpoint blockade	MSLN	αPD1 antibodies secretion	MSLN + advanced malignancies	–	Phase I/II NCT03030001	Unknown status	–	–
	MSLN	αPD1 antibodies secretion	MSLN + solid malignancies	Apatinib (anti-angiogenic drug)	Phase I/II NCT03615313	Unknown status (50 to include)	1 advanced/refractory OC infused patient: 1PR and survival > 17 months	(316)
	MSLN	αPD1 nanobodies secretion	NSCLC Mesothelioma	Lymphodepletion (cyclophosphamide)	Early Phase I NCT04489862	Recruiting	None posted	–
	MSLN	αPD1 nanobodies secretion	Advanced CC and OC	Lymphodepletion (cyclophosphamide)	Early Phase I NCT04503980	Recruiting	None posted	–
	MSLN	αPD1 nanobodies secretion	CC	–	Phase I NCT05089266	Not yet recruiting	None posted	–
	–	αPD1 antibodies expression	EGFR+ solid tumors	–	Phase I/II NCT02873390	Unknown	–	–
	MUC-1	CTLA-4 and PD-1 antibodies expression	MUC1+ Advanced Solid Tumors	Cyclophosphamide	Phase I/II NCT03179007	Unknown	None posted	–
	EGFR	CTLA-4 and PD-1 antibodies expression	EGFR + advanced recurrent/refractory solid tumors	Cyclophosphamide	Phase I/IINCT03182816	Unknown	9 NSCLC:1/9PR (lasted> 13m),6/9 SD and 2/9 PD, no grade 4 AEs, Grade 1-3 AE	(317)
	MSLN	CTLA-4/PD-1 antibodies expression	Advanced solid tumors	Cyclophosphamide	Phase I/IINCT03182803	Unknown	None posted	–
	HER2, MSLN PSCA, MUC-1, Lewis-Y, GPC3, AXL, EGFR, Claudin18.2/6, ROR1, GD1, or B7-H3	CRISPR-Cas9 PD1 KO and CTLA-4/PD-1 -scFv secretion	Solid tumors	–	Phase I NCT04842812	Recruiting	None posted	–
	PSCA, MUC1, TGFβ, HER2, MSLN, Lewis-Y, GPC3, AXL, EGFR, B7-H3, Claudin18.2	CD4+: TGFβ-CAR and expression and secretion of IL7/CCL19 and/or SCFVs against PD1/CTLA4/TIGITCD8+: PD1 KO	Lung cancer	–	Phase INCT03198052	Recruiting	None posted	–
Protection from IS	PD-L1	PD1/CD28 chimeric switch receptor truncated EGFR (tEGFR) for CSR ablation	Glioblastoma	Cyclophosphamide and Fludarabine)	Phase I NCT02937844	Unknown	None posted	–
	PSMA	Non-viral PD-1integration	CRPC	Cyclophosphamide and Fludarabine	Phase INCT04768608	Not yet recruiting	None posted	–
	MSLN	CD3z signaling domain with loss-of-function mutations within 2 of 3 ITAM motifs and PD-1 dominant-negative receptors (DNR)	Mesothelioma	Cyclophosphamide	NCT04577326	Recruiting	None posted	–
	PSMA	Dominant negative TGFβ receptor (TGFβDNR)	Advanced CRPC	+/- Cyclophosphamide	Phase INCT03089203	Active, not recruiting	4/10 reached PSA30 response, 5/10 CRSgrade ≥ 2, 1/10grade 5 AE	–
	PSMA	TGFβDNR	Metastatic CRPC	Fludarabine & CyclophosphamideAnakinra	Phase INCT04227275	Active, not recruiting	None posted	–
	CD22	anti-PD-L1 scFv	Cervical cancer, sarcoma, NSCLC	–	Phase 1NCT04556669	Recruiting	None posted	–
CAR-T cell maintenance	GD2	Constitutively active IL-7 Receptor	GD2+ brain cancers (HGG, DIPG, medulloblastoma)	Lymphodepleting chemotherapy (Cyclophosphamide & Fludarabine)	Phase INCT04099797	Recruiting	None posted	–
	GPC3	IL15 & IL21armoredInducible caspase 9 safety switch (iCas9)	Pediatric solid tumors*	Lymphodepleting chemotherapy (Cyclophosphamide &Fludarabine)	NCT04715191	Not yet recruiting	None posted	–
	GPC3	IL15 & IL21armored	HCC/Hepatoblastoma	–	NCT04093648	Withdrawn/incorporated into another study	None posted	–
	GPC3	IL15 armored	Pediatric solid tumors	–	Phase INCT04377932	Recruiting	–	–
	GPC3	IL15 armored	HCC	–	Phase INCT05103631	Not yet recruiting	–	–
	GPC3	ArmoredCAR-T cells	Advanced HCC	–	Phase INCT05155189	Not yet recruiting	None posted	–
	GPC3	Expression of IL-21 and/or IL-15	Pediatric Liver Tumors	Cyclophosphamide & Fludarabine	Phase I NCT02932956	Recruiting	None posted	(318)
	GPC3	Expression of IL-21 and/or IL-15	HCC	Cyclophosphamide & Fludarabine	Phase INCT02905188	Recruiting	None posted	–
	GD2	iCas9 safety switch,expression of IL-15	R/R neuroblastoma and osteosarcoma	Cyclophosphamide & Fludarabine	Phase INCT03721068	Recruiting	None posted	–
Combinatorial strategies	GPC3	–	HBV-related metastatic HCC	Lymphodepletion+ Tyrosine kinase inhibitors (TKIs) sorafenib/regorafenib (in 67%) +/- PD-1/PD-L1 mAb	Phase I NCT03980288	6 patients	1 PR persisting at 18 months	(319)

Registered trials listed in the table were either from www.clinicaltrials.gov or from www.Chictr.org.cn (Chinese Clinical Trial Registry, marked with **).

AE, adverse effects; ALL, Acute Lymphoblastic Leukemia; BCMA, B-cell maturation antigen; CLL, Chronic Lymphocytic Leukemia; CC, colorectal cancer; CR, complete response; CRPC, Castration Resistant Prostate Cancer; CRS, Cytokine release syndrome; CTCL, Cutaneous T-Cell Lymphoma; RR DLBCL, relapsed or refractory diffuse large B cell Lymphoma; DIPG, diffuse intrinsic pontine glioma; FAP, Fibroblast activation protein; GPC3, glypican-3; HCC, Hepatocellular Carcinoma; HGG, high grade glioma; HL, Hodgkin Lymphoma; Hsvtk, herpes simplex virus-1 thymidine kinase; Interleukin, IL; IS, immunosuppression; KO, Knock-out; mAb, monoclonal antibody; MM, Multiple Myeloma; MRCL, Myxoid/Round Cell Liposarcoma; MSLN, mesothelin; NGFR, Nerve Growth Factor Receptor; NHL, Non Hodgkin Lymphoma; NSCLC, non-small-cell lung carcinoma; OC, ovarian carcinoma; OS, overall survival; ESCC, esophageal squamous cell carcinoma; PC, pancreatic cancer; PD, progressive disease; PFS, progression-free survival; PR, partial response; PSCA, Prostate stem cell antigen; rhIL2, Recombinant Human IL-2; RR BCL, relapsed/refractory B cell lymphoma; RR MM, refractory/recurrent multiple myeloma; scFv, single chain variable region; SD, steady disease; SS, Synovial Sarcoma; VGPR, very good partial response.

A different strategy to provide an enhanced version of genetically modified T cells with increased antitumor activity against solid tumors was to engineer PD-1 knockout (KO) CAR-T cells. The breakthrough of gene editing techniques has, thus, allowed to genetically disrupt PD-1 function in CAR-T-cells (320). As a strategy that increases lymphocyte fitness in the immunosuppressive TME, PD-1 KO engineering in CAR-T cells proved enhanced antitumor activity in preclinical models, both *in vitro* and *in vivo* (283, 321). PD-1 deletion was first applied to autologous T cells and its efficacy was variable, depending on gene editing techniques. To this regard, Beane et al. used the zinc finger nuclease-mediated (ZFN) gene editing technology to KO PD1 expression in TILs drawn from melanoma patients, with an average reduction of 76% in PD-1 surface-expression. In addition, PD-1 KO TILs showed enhanced *in vitro* activity and a significantly superior polyfunctional cytokine profile (IFNγ, TNFα, and GM-CSF) as compared to unmodified TILs in 66.67% (2 of 3) patients tested (322). Menger et al. knocked out the PD-1 gene in melanoma-reactive CTLs and in fibrosarcoma-reactive polyclonal T cells, using the TALEN technology and noticed that modified T cells had better persistence at the cancer site and were able to control the tumor progression more efficiently than non-modified T cells (323). Other groups have also succeeded in inactivating the PD-1 gene in TILs by using the CRISPR-Cas9 technology (321, 324, 325).

Considering immune-related adverse events related to anti-PD-1 mAbs administration and the success of PD-1 gene inactivation in primary human T cells, this strategy was extended to CAR-T cell therapy (326). Hu B. et al., designed CAR-T cells directed against CD133 and KO for the PD-1 gene by using the CRISPR/Cas9 technology, with an average of 91.5% of inactivated gene sites. This disruption enhanced both *in vitro* cytotoxicity against a glioma cell line and *in vivo* antitumor activity in an orthotopic glioma mouse model. No significant toxicity was observed, confirming the safety profile of PD-1 KO CD133-specific CAR-T cells. Moreover, PD-1 KO did not impede cytokine production and CAR-T functionality as PD-1-deficient lymphocytes secreted similar amounts of cytokines (IFN-γ, IL-2, TNF-α, and GM-CSF) as conventional CAR-T cells (286). The team used this technology to disrupt the PD-1 gene in MSLN-specific CAR-T cells also. Despite a significant effect on CAR-T cell proliferation, this strategy greatly increased CAR-T cell cytokine synthesis and cytotoxicity towards PD-L1+ tumor cells *in vitro*. The antitumor activity of PD-1 KO MSLN-specific CAR-T cells was also increased in a TNBC animal model. Moreover, PD-1 KO could improve the CAR-T cells antitumor effect more efficiently than the combination of CAR-T cells with PD-1 blocking (287). Similar results have been published by Guo et al. with PD-1 KO GPC3-specific CAR-T cells in a HCC preclinical study (288), and Choi et al. with PD-1 KO EGFRvIII-specific CAR-T cells in a glioblastoma preclinical study (289). Many ongoing clinical trials are evaluating the efficacy of PD-1 deficient T cells/CAR-T cells in patients with solid tumors or hematological cancers (NCT03545815, NCT03525782, NCT03298828, NCT03525652, NCT02793856, NCT03081715, NCT03399448, NCT02867332, NCT03044743, NCT03030001, NCT02863913, NCT03747965, NCT03706326, ChiCTR1800020306, ChiCTR1800018713, ChiCTR1900022620, ChiCTR-OIC-1701131, ChiCTR-OIN-17012136, ChiCTR1800016023, ChiCTR1900025088, NCT03208556 and NCT04213469) (**Table 10** for clinical tials on solid tumors**)**.

Last but not least, new reports have suggested the feasibly of targeting other inhibitory receptors, such as CTLA-4 (327, 328), LAG-3 (329, 330) or TIM-3 (330, 331). However, additional studies are required to determine whether these novel strategies are as effective as CAR-T cells engineered to overcome PD1 inhibition. Results from ongoing clinical trials with CAR-T engineered to block simultaneously PD-1 and CTLA-4 +/- TIGIT (by antibody or ScFv secretion) will establish the eventual benefit of combinatorial ICI blockade strategies (NCT03179007, NCT03182816, NCT03182803, NCT04842812, NCT03198052).

## 3 Conclusion and Future Perspectives

CAR-T cell based cell therapy is a moving field, which showed impressive results in hematopoietic cancer management and brought hope to incurable patients. Unfortunately, success in managing solid cancers was less outstanding. Assiduous research has been done to overcome unexpected roadblocks which impede CAR-T cells trafficking, infiltration, persistence or function in the unwelcoming tumor environment. Indeed, research focused on identifying target antigens and avoiding on-target-off tumor toxicity (206), improving CAR-T cell trafficking and entry into the tumor site, promoting better signaling, less exhaustion, and memory phenotypes in solid tumors. Preclinical models propose various engineering strategies, some of which have already advanced from bench to bedside, with encouraging preliminary results.

As reviewed herein, trafficking and infiltration have been addressed by genetically manipulating chemotaxis and tissue homing. Moreover, tumor stroma targeting emerged as a promising strategy, based either on depletion of stromal cells/immunosuppressive cells or at reprogramming strategies directed at regulating TME plasticity. To this regard, a new generation of CAR-T cells has been designed to directly target stroma components like fibroblasts and immunosuppressive cells (Tregs, TAMs or MDSCs). However, a remaining challenge for the development of both effective and safe CAR-T cell therapies is the insufficient clinical relevance of preclinical mouse models. Indeed, these models sometimes failed to predict clinical level toxicities or, on the other hand, inefficient tumor targeting when translated to the clinic. Further research is still needed to overcome this hurdle and develop advanced preclinical models able to address tumor heterogeneity and TME complexity in order to accomplish a perfect balance between efficacy and safety of CAR-T cell therapies in solid tumors.

Furthermore, exciting new opportunities emerged thanks to gene editing/gene ablation techniques based on the revolutionary, highly specific and efficient CRISPR/Cas9 tools (332, 333), which have been used not only to generate immune-checkpoint knock-outs (PD-1 KO) but also to design “universal” CARs, edited for TCR and/or HLA molecules expression (206, 332, 333), which could pave the road towards cost-effective allogeneic CAR-T cells for an “off-the-shelf” ACT with a broader spectrum (334, 335). This technique can even be used for multiplexed genome editing (336). To this regard, feasibility of targeting multiple genes in T cells by multiplex CRISPR-Cas9 has recently been proven in a small interventional study in patients with advanced, refractory cancer (NCT03399448) (312). Further improvements of this technology are awaited as recent advances seem to insure increased precision and minimized side effects both in case of gene deletion and gene insertion (336). As allogeneic (allo)-CAR-T cells could offer readily available ACT sources that could expand the usage of CAR-cells based immunotherapy, other recent strategies for allo-CAR-T cells generation emerged, like the NKG2D (an NK-based activating receptor) expression. NKG2D expression in allogeneic CAR-T cells offers a non-TCR edited cellular therapy with broad solid tumor targeting, and two clinical trials are ongoing in metastatic colon cancer (NCT04991948 and NCT03692429, Celyad Oncology) with encouraging preliminary results in the second one (2/15 PR and 9/15 SD). Another allo-CAR-T cells product, the CD70-targeting ALLO-316 cells (Allogene Therapeutics) is under evaluation in a clinical trial on renal cell carcinoma (together with anti-CD52 mAb, NCT04696731).

On the other hand, a part from innovations in CAR design addressed in this review, advances in transduction techniques, cell culture and amplification conditions (like IL-7/IL-15 media) as well as identification of the most suitable stage of T cell differentiation (TCM/TSCM) to use for adoptive transfer represent additional steps towards effective CAR-T cell therapy in solid tumors (337, 338). To this regard, the need for large-scale CAR-T manufacturing persists and could limit cancer patients’ accessibility to CAR-T cell-based ACT. Therefore, the already engaged transition from academic to industrial manufacturing could ensure increased availability and reproducibility as well as shorter delays thanks to Good Manufacturing Practice (GMP)-compliant automated, closed systems (339–341). Contrary to large scale, commercial *in vitro* manufacturing, Smith and colleagues recently described an *in-vivo* manufacturing technique of CAR-T cells, by programming circulating, bloodstream T cells with DNA-carrying polymer nanoparticles, which efficiently introduced leukemia-targeting CAR genes into T-cell nuclei (342, 343).Accumulating knowledge on efficacy, toxicity and resistance drawn from clinical trials as well as fundamental research data on TILs interaction with the TME will allow for the identification of novel molecular targets in CAR-T cells design (344). To this regard, and pointing out once more the role of the hypoxia response in cancer, the VHL-HIF axis and particularly HIF’s activity, has recently been identified as a tool to potentiate tissue residency of CD8+ CTLs, as well as a potential molecular candidate to modulate CAR-T cell therapy efficacy (345). Genetic targeting of precise molecular or metabolic pathways critical for TILs survival in the TME emerge therefore as novel strategies to overcome insufficient amplification and persistence of CAR-T cells in solid tumors (31, 213, 346, 347).

This review focuses less on engineering strategies aiming at enhancing tumor recognition and preventing antigen escape. Such combinatorial targeting strategies employ bispecific/dual CARs or Tandem CARs, trivalent or pool CARs and have already been reviewed by us (206) and others (31, 213, 346, 348). Bispecific CARs have gained an important place in hematologic cancers management, with numerous ongoing clinical trials (NCT04662099, NCT0327115, NCT03919526, NCT03879382, NCT03881761, NCT03706547, NCT04303520, NCT04412174, NCT03825731, NCT04499573, NCT05098613, NCT04034446, NCT04007029, and NCT04215016). However, usage of bispecific CARs in solid tumors is still at its beginning (NCT03672305, NCT04483778, NCT03618381 and NCT04684459) (**Table 10**). Nonetheless, multiple antigen targeting by employing universal immune receptors CAR also gains increasing interest (349) and a universal CAR is being tested in a clinical trial on prostate cancer patients (UniCAR02, NCT04633148). Moreover, toxicity management strategies and especially prevention of on-target off tumor effects were not thoroughly described here-in but were reviewed previously (206). Switchable CARs for instance emerge as valuable safety strategies, like is the case of the iCas9 safety suicide switch employed in some ongoing clinical trials (NCT04715191, NCT03721068). Moreover, orthogonal switchable CARs or dual-switch CAR-T cells capable of both regulated costimulation/inducible activation to drive CAR-T cell expansion and activity and regulated iCasp9 safety switch for CAR elimination have recently been described (350). Co-activated switchable CAR-T cells also advanced to clinical testing (ongoing clinical trial NCT02744287 of BPX-601 CAR-T cells expressing a PSCA specific CAR and a rimiducid-inducible MyD88/CD40 co-activation switch, Bellicum Pharmaceuticals), with encouraging preliminary results (307) (**Table 10**).

Besides optimization of costimulatory domains discussed here-in, modulation of scFv avidity could be another strategy to increase antigen recognition and CAR-T cell engagement. Surprisingly, lower avidity CAR-T cells (the 8F8-BBz CAR-T cells) could show greater therapeutic potential, by increased resistance to exhaustion and apoptosis in an HCC context (351).

Considering all the aforementioned hurdles in CAR-T cells homing, as well as the diversity and plasticity of cells composing the tumor microenvironment, the best engineering option could be based on a combination of strategies that enhance at the same time trafficking, penetration, persistence and/or CAR-T cell function. Some combinations have already been tested, like it is the case of armored CAR-T cells/TRUCKs engineered to co-express chemokine receptors and secrete vital cytokines. As monotherapeutic approaches are rarely effective, strategies targeting multiple antigens, combinations of different genetic engineering strategies or combinations based on CAR-T cells and innovative immunotherapies (like ICIs) could represent a turning point in a still ongoing revolution in solid cancer management. Nonetheless, CAR-T cells could also be combined with other therapeutic modalities, such as standard chemotherapy and/or radiation therapy, tyrosine kinase inhibitors, epigenetic modulators, other small molecule drugs or vaccines.

All in all, CAR-T cell immunotherapy stands out as a promising, evolving weapon in the fight against solid cancer.

Beside CAR-T cell based ACT, novel genetic engineering techniques, such as gene-editing and cellular reprogramming allowed for the emergence of new ACT strategies employing various innate killer cells (IKC) (like NK cells, NKT cells, and γδ T-cells (CAR-IKC) (352–358), macrophages (CAR-M) (146), and even B lymphocytes (CAR-B cells). A combination of “classic” CAR-T cells and CAR- IKC/CAR-Macrophages as bridging therapy could potentially increase efficiency in solid tumors by increasing the cross-talk between various immune cells or by TME remodeling effects (248, 359, 360). Unfortunately, CAR-NK also have some limitations, as for example high-dosage conditioned efficacy and decreased persistence. On the other hand, co-administration of cord-blood derived-NK cells (CB-NKs) proved to be a potent immunoregulatory strategy, promoting early activation and migration, enhanced fitness and increased anti-tumor efficacy of CAR-T cells (360). Surprisingly, chimeric receptor engineered Tregs (CAR-Tregs), which emerged as potential immune-tolerance inducers in autoimmunity or transplantation (361), also showed potent anti-tumor effect (362). Moreover, CAR-B cells, which could represent safe and controllable vehicles for local delivery of monoclonal antibodies emerged in preclinical studies as potential candidates for infectious diseases and protein deficiencies and might therefore be interesting candidates for cancer therapy as well.

## Author Contributions

AA and AC contributed equally. All authors listed have made a substantial, direct, and intellectual contribution to the work, and approved it for publication.

## Conflict of Interest

The authors declare that the research was conducted in the absence of any commercial or financial relationships that could be construed as a potential conflict of interest.

## Publisher’s Note

All claims expressed in this article are solely those of the authors and do not necessarily represent those of their affiliated organizations, or those of the publisher, the editors and the reviewers. Any product that may be evaluated in this article, or claim that may be made by its manufacturer, is not guaranteed or endorsed by the publisher.
